# Resistance haplotypes to green rice leafhopper (*Nephotettix cincticeps* Uhler) estimated in genome-wide association study in Myanmar *indica* rice landraces

**DOI:** 10.1270/jsbbs.23067

**Published:** 2024-08-23

**Authors:** Nang Moe Kham, Hiroyuki Kanamori, Jianzhong Wu, Takashi Matsumoto, Daisuke Fujita, Hideshi Yasui, Atsushi Yoshimura, Yoshiyuki Yamagata

**Affiliations:** 1 Plant Breeding Laboratory, Faculty of Agriculture, Kyushu University, 744 Motooka, Nishiku, Fukuoka 819-0395, Japan; 2 Institute of Crop Science, National Agriculture and Food Research Organization, 2-1-2 Kannondai, Tsukuba, Ibaraki 305-8518, Japan; 3 Department of Bioscience, Faculty of Life Sciences, Tokyo University of Agriculture, 1-1-1 Sakuragaoka, Setagayaku, Tokyo 156-8502, Japan; 4 Faculty of Agriculture, Saga University, 1 Honjo-machi, Saga 840-8502, Japan

**Keywords:** rice, green rice leafhopper, resistance, haplotype, marker-trait association

## Abstract

Green rice leafhopper (GRH, *Nephotettix cincticeps* Uhler) is a serious insect pest of rice in the temperate regions of Asia. Myanmar has a high genetic diversity and is located at the center of the origin of rice. To understand the genetic architecture of GRH resistance in Myanmar *indica* rice landraces, a genome-wide association study (GWAS) was performed using a diversity panel collected from diverse geographical regions. Phenotypic variation in GRH resistance was associated with three genomic regions, MTA4, MTA5, and MTA11, located on chromosomes 4, 5, and 11, respectively. MTA4 and MTA5 were adjacent to the known resistance genes *GRH6* and *GRH1*. Analysis of haplotypes and linkage disequilibrium blocks revealed that the haplotypes Hap*GRH6*A, Hap*GRH1*A, and HapMTA11A mainly explained GRH resistance. Map-based cloning revealed that *GRH6* was highly induced by GRH infestation and conferred resistance by inhibiting the sucking of phloem sap. The distribution of resistance haplotypes revealed that accessions harboring major resistance haplotypes (Hap*GRH6*A and Hap*GRH1*A) were mainly distributed in Southern Myanmar, and HapMTA11A was mainly responsible for GRH resistance in mountainous areas of Myanmar. Our findings could facilitate the elucidation of the molecular mechanism of GRH resistance and provide essential haplotype-based genetic information for the development of GRH-resistant rice cultivars.

## Introduction

The green rice leafhopper (GRH, *Nephotettix cincticeps* Uhler) is a significant insect pest of cultivated rice (*Oryza sativa* L.) in the temperate regions of Asia (Ghauri 1971). GRH damages rice plants by sucking sap from the xylem and phloem of susceptible rice cultivars, thereby reducing yield (Pathak and Khan 1994). Additionally, GRH transmits viral diseases, such as rice dwarfs (Brar *et al.* 2009). Among various control measure, utilization of resistant rice varieties is the most cost effective and efficient strategy to mitigate insect pest infestation. Therefore, the exploration of insect resistance genes in rice and development of resistant rice cultivars have become focal point for breeders (Yan *et al.* 2023). To date, six genes and two quantitative trait loci (QTLs) conferring resistance to GRH have been identified in cultivated rice and its wild relatives. These resistance genes include *GRH1* on chromosome 5 in the cultivars Pe-bi-hun, IR24, Singwang, and ASD7 (Kadowaki *et al.* 2003, Mai *et al.* 2015, Park *et al.* 2013, Tamura *et al.* 1999), *GRH2* on chromosome 11 and *GRH4* on chromosome 3 in the cultivars Lepe-dumai and DV85 (Fukuta *et al.* 1998, Kadowaki *et al.* 2003), and *GRH3* on chromosome 6 in the cultivars Rantaj-emas 2 and Cheongnam (Hur *et al.* 2015, Saka *et al.* 2006). *GRH6* has been mapped to the short arm of chromosome 4 in the Surinam cultivar SML17 (Tamura *et al.* 2004) and a wild accession of *O. nivara* IRGC105715 (Fujita *et al.* 2004). *GRH5* was detected on chromosome 8 in *O. rufipogon* Griff. accession number W1962 (Fujita *et al.* 2006). A minor QTL, *qGRH4*, was discovered in the backcross population between the *O. sativa* ssp. *japonica* cultivars Taichung 65 and W1962 (Fujita *et al.* 2006). A major QTL, *qGRH9*, was identified on the long arm of chromosome 9 in the African species *O. glaberrima* (Fujita *et al.* 2010). Some GRH resistance genes derived from landraces and wild relatives have been introduced to elite genetic background of rice cultivars such as Norin PL2 (*GRH1*) (Kaneda *et al.* 1985), Aichi 80 (*GRH3*(t)) (Nakagome *et al.* 1989), Norin PL5 (*GRH1* and *GRH2*) (Takita 1990), Norin PL6 (*GRH2* and *GRH4*) (Fukuta *et al.* 1998). However, with the emergence of new insect biotypes, the resistant cultivars have either lost or gradually reduced their resistance (Pathak and Heinrichs 1982). The GRH populations from paddy field at Hokuriku Research Center were virulent against rice varieties with *GRH1* and *GRH2* (Hirae *et al.* 2007). Therefore, screening of large scale germplasm for GRH resistance, and exploration of resistance genes from these genetic resources play crucial role for development of durable GRH resistant varieties.

With the rapid development of genome sequencing technologies, genome-wide association studies (GWAS) have emerged as a potent strategy for identifying QTLs in various crop germplasms using high-density SNP genotypes and accurate phenotypic data (Dhatt *et al.* 2021, Zhao *et al.* 2011). Over the past several years, GWAS have effectively identified quantitative trait loci (QTLs) associated with traits such as abiotic and biotic stress resistance in rice (Dimkpa *et al.* 2016, Fujino *et al.* 2015, Kang *et al.* 2016, Shi *et al.* 2021, Zhang *et al.* 2019). The mixed-model GWAS approach has been widely used to control false positives (type I errors) caused by the population structure and relatedness of the panel (Kang *et al.* 2008). The mixed model involved fixed effects of additive SNP effects and population structure and random effects due to polygenic effects in the genetic background of lines defined as a kinship covariance matrix (Yu *et al.* 2006). The fixed and random effects were simultaneously estimated as the Best Linear Unbiased Estimates (BLUEs) and Best Linear Unbiased Predictions (BLUP) by solving a mixed-model equation (Henderson 1975). The genomic best linear unbiased prediction (GBLUP) uses a genomic relationship (GRM) matrix estimated from genome-wide SNP as an approximation of the kinship matrix (Hayes *et al.* 2009). The haplotype-based GWAS approach is more efficient for detecting genomic loci and candidate genes for the desired traits (Hamazaki and Iwata 2020). A haplotype consists of closely linked genomic structural variations, such as polymorphic SNPs, with strong linkage disequilibrium among them (Maldonado *et al.* 2019). Individual SNP markers are biallelic and are poorly informative (Würschum *et al.* 2013). Significant SNPs often do not represent causal molecular variants (Bhat *et al.* 2016, Zargar *et al.* 2015). The multi-allelic nature of haplotype blocks is more informative than that of SNP markers due to the abundance of haplotype variants that indicate recombination and mutation events within genes (Stephens *et al.* 2001). Compared with individual SNP markers, haplotypes provide more accurate predictions of genomic regions associated with low-heritable quantitative traits (Calus *et al.* 2009).

Many resistance genes are clustered in the plant genome, likely due to tandem duplications. They also exhibit high levels of inter- and intraspecific variation in copy number, owing to mechanisms such as unequal crossing-over, sequence exchange, and gene conversion (Kuang *et al.* 2004). Four notable gene clusters contained several brown planthopper (BPH) resistance genes: clusters A, B, C, and D on chromosome 12, the short arm of chromosome 4, chromosome 6, and the long arm of chromosome 4. Genes resistant to sucking insect pests of rice including *Glh14*, *Grh6-nivara*, *Bph15*, *Bph17*, *Bph20* and *Bph12* (Fujita *et al.* 2004, Qiu *et al.* 2012, Rahman *et al.* 2009, Sebastian *et al.* 1996, Sun *et al.* 2005, Yang *et al.* 2004) are found within cluster B on the short arm of chromosome 4. Some resistance loci contain only one gene with multiple distinct alleles (Ronald 1998). Resistance genes containing the nucleotide-binding site leucine-rich repeat (NBS-LRR) domains are more abundant in Asian cultivated rice (*O. sativa* L.) than in their wild ancestors because of the tandem duplication of chromosomal segments during domestication and repeated cultivation of specific genotypes (Mizuno *et al.* 2020).

Most of the previously cloned resistance genes (R genes) in plants encode proteins with NBS-LRR domains. These proteins recognize pathogen effectors and trigger defense responses (Lee and Yeom 2015). Genes encoding NBS-LRR proteins are prevalent in plants; for example, there are 149 and 500 NBS-LRR family genes in the genomes of Arabidopsis and rice, respectively (Wang *et al.* 2023). Effector-triggered immunity in plants activates a series of defense-related signaling pathways, which leads to the accumulation of substances, such as callose, in sieve tubes. These changes, including cell-wall thickening, hinder BPH feeding, growth, and reproduction (Yan *et al.* 2023).

Rice defense responses usually involve the activation and expression of numerous defense genes. Transcription factors play a pivotal role in regulating the transcription of defense-associated genes (Chang *et al.* 2023). Several pathogen-inducible genes have been identified, including those for salicylic acid (SA), jasmonic acid (JA), and ethylene (ET). This demonstrates the significance of the interconnected signaling pathways mediated by these phytohormones in rice defense against pathogens (De Vleesschauwer *et al.* 2013). The transcription factors WRKY and MYB are vital for orchestrating rice defense responses by activating the transcription of genes responsible for the biosynthesis and signaling of these phytohormones (https://doi.org/10.1101/2022.12.12.520141, Eulgem *et al.* 2000).

In this study, we investigate the genetic architecture of GRH resistance in the diversity panel of Myanmar *indica* rice landraces in GWAS. Haplotype estimation inferred the haplotypes associated with GRH resistance at all detected loci. Map-based cloning revealed the causative gene and resistance-associated haplotype at *GRH6*. The geographical distribution of GRH resistant haplotype combination in Myanmar *indica* landraces was investigated to understand the genetic composition for GRH resistance in this population in response to the different geographical areas.

## Materials and Methods

### Plant materials

From the Seedbank of the Department of Agricultural Research, Ministry of Agriculture, Livestock, and Irrigation, Myanmar, 464 accessions of the representative collections, which had been selected from phenotypic variation and different geographical origins in Myanmar, were used to investigate their population structure by the genotyping-by-sequencing (Furuta *et al.* 2024). Among the observed three major clusters, which seemed corresponded to the so-called *japonica*, *indica* and aromatic groups, the 250 *indica* accessions were selected and designated the Myanmar *indica* diversity panel ‘MIDP’. Whole genome sequencing and variant analysis showed that the MIDP populations were apparently distinguished from the representative accessions from the aus/boro subpopulation in 3000 genome project and the no apparent subpopulation structure within the MIDP (Furuta *et al.* 2024). From the 250 accessions of MIDP, only 224 accessions were available for evaluation of GRH resistance.

The resistant *indica* rice cultivar DV85, which carries the resistance genes *GRH2* and *GRH4*, and the susceptible *japonica* cultivar Taichung 65 (T65) were used as controls in the antibiosis test. A near-isogenic line harboring *GRH6* derived from the *O. nivara* accession WK56 (*GRH6-WK56* NIL) was used as a resistant control in the transformation experiment for map-based cloning. *GRH6-WK56* NIL was developed from the *O. nivara* (Fujita *et al.* 2004) accession WK56 (IRGC105715), the donor of *GRH6-nivara* (Fujita *et al.* 2004), by continuous backcrossing to the recurrent parent T65 (Fujita *et al.* 2010). The WK56 accession was isolated at Kyushu University, Fukuoka, Japan, through successive self-pollination using the accession IRGC105715 obtained from the International Rice Research Institute, Los Baños, Philippines.

### Antibiosis test for GRH resistance

The GRH strain used in this study was collected in Fukuoka, Japan, in 1990 and has been maintained by continuously rearing on seedlings of the susceptible *japonica* cultivar Nipponbare under conditions of 25°C with 16 hour of light and 8 hour of darkness. An antibiosis test was conducted to evaluate the resistance to GRH. Ten-day-old rice seedlings were infested with ten first-instar GRH nymphs in test tubes with five replicates per accession. Nymph mortality (%) for each accession was determined at 3, 5, and 7 days after infestation (DAI). Accessions exhibiting nymph mortality rates of less than 30%, 30 to70%, and greater than 70% were categorized as susceptible (S), moderately resistant (MR), and highly resistant (HR) classes, respectively. The resistance classes at 7DAI determined the final categorization of the resistant phenotypes of the cultivars.

### Genome-wide association study

Whole genomic sequences of the diversity panel were determined with DNA nanoball-sequencing (DNB-seq) technologies. Cutting off of the bases with the low-quality score were conducted with the Trimmomatic software (Bolger *et al.* 2014). The qualified pair-end reads were mapped on Nipponbare reference sequence (Os-Nipponbare-Reference-IRGSP-1.0, Kawahara *et al.* 2013) using the bwa-mem software (https://doi.org/10.48550/arXiv.1303.3997). The variant call procedure follows the best practice workflow of the germline short variant discovery of the Genome analysis tool kit version 4 (GATK4) software (https://doi.org/10.1101/201178). The obtained variants were selected by hard filtering conditions; “QualByDepth (QD) < 2.0” --filter-name “QD2” -filter “Phred-scaled probability (QUAL) < 30” --filter-name “QUAL30” -filter “StrandOddsRatio (SOR) > 3.0” --filter-name “SOR3” -filter “FisherStrand (FS) > 60.0” --filter-name “FS60” -filter “RMSMappingQuality (MQ) < 40.0” --filter-name “MQ40” -filter “MappingQualityRankSumTest (MQRankSum) < -12.5” --filter-name “MQRankSum-12.5” -filter “ReadPosRankSumTest (ReadPosRankSum) < -8.0” --filter-name “ReadPosRankSum-8”. Using the vcftools software (Danecek *et al.* 2011), the variants were filtered with a minor allele frequency (maf) of more than 0.05 and genotype missing frequency of less than 0.05.

GWAS using GBLUP was conducted using the R package rrBLUP (Endelman 2011). The genomic relationship matrix was computed from the 256,347 SNP loci randomly selected across the 12 chromosomes using the A.mat () function. A –log_10_(*p*) value exceeding five was deemed significant for associations. Marker-trait associations (MTAs) were identified as the SNPs most significantly associated with a minor allele frequency over 5% and in more than 95% of individuals genotyped the nymph mortality of GRH. The linkage disequilibrium (LD) block containing significant MTAs was discerned using LD heat maps constructed with the R/LD heat map software (Shin *et al.* 2006). As the Myanmar diversity panel comprises pure lines of landrace varieties, estimating the dominance effect (*d*) at an SNP site is impossible, setting *d* to zero. Consequently, the fixed effect of the breeding value at an SNP site was assumed to be equal to the additive effect of the major allele.

### Haplotype analysis

A genomic haplotype was characterized by a combination of nucleotide variations at multiple SNPs with highest –log_10_(*p*) value within the fine mapping region of QTL or genomic region associated with the GRH resistance. For haplotype analysis in candidate gene, the unfiltered genotyped data was considered to access all variants of the SNPs and InDel calls. After filtering the SNPs and InDels with a minor allele frequency (maf) of more than 0.05 and genotype missing frequency of less than 0.1, the high quality SNPs and InDels in coding sequence (CDS), intron and untranslated regions (UTR) were used for haplotype variation analysis. Haplotypes with frequencies exceeding 0.05 were considered major haplotype variants and the remaining ones were regarded as unknown haplotypes.

### QTL analysis

To validate the MTA11 detected in GWAS, QTL analysis was conducted in the bi-parental mapping population (F_2_) derived from the cross between CC125, the Myanmar accession carrying the resistance haplotype at MTA11 (HapMTA11A) and T65. GRH nymph mortality of 77 F_2_ individuals was evaluated at 3DAI using antibiosis test at the seedling stage of rice. Total genomic DNA of each F_2_ plants was extracted by potassium acetate method (Dellaporta *et al.* 1983). To develop the InDel markers around the MTA11 region, whole genome variant information of the CC125 and T65 were combined with the GATK4/GenotypeGVCFs command and their InDel variants were called with GATK4/GenotypeGVCFs. Genotyping was conducted using six InDel markers covering the MTA11 region for the QTL confirmation (Supplemental Table 1). The PCR reaction contained an amount of approximately 10 ng of genomic DNA, 0.2 μM primer, and 7.5 μl of GoTaq Master mix (Promega, Fitchburg, WI, USA). The thermal profile included an initial denaturation at 95°C for 5 min, 35 cycles of 95°C for 30 sec and 72°C for 30 sec, and a final elongation step at 72°C for 7 min. The amplified products were visualized in a 4% agarose gel by electrophoresis at 250 V for about 45 min in 0.5xTBE buffer. The gels were stained by ethidium bromide and photographed under ultraviolet light. QTL analysis was performed using R/qtl (Broman and Sen 2009). The marker regression was used to estimate genetic effect and logarithm of odds (LOD) score. The LOD threshold more than 1% level of significant was defined as a significant QTL. LOD threshold was calculated from 1000 permutation test.

### Complementation test of *GRH6*

Genomic DNA obtained from *O. nivara* bacterial artificial chromosome (BAC) clone IRGC105715-26E15 containing the candidate gene of *GRH6*, *Os04g0166000* was digested with the restriction enzyme *Avr*II. The resulting digests were subcloned into the Ti plasmid binary vector pPZP2H-*lac* (Fuse *et al.* 2001) and introduced into the susceptible T65 cells via *Agrobacterium*-mediated transformation (Toki 1997). The resistance to GRH in sixteen T_0_ plants containing *Avr*II fragment and five T_0_ plants with empty vector was evaluated using antibiosis test at the seedling stage. The nymph mortality (%) was evaluated at 3, 5 and 7DAI.

### Expression analysis

Twelve-days old rice seedlings of T65 and *GRH6-WK56* NIL were infested with GRH. Roughly 3 cm of leaf blade from non-infested and infested seedlings were collected at 0, 1, 2 and 3DAI with 3 replications. The leaf samples were instantly frozen in liquid nitrogen at each DAI and stored at –60°C prior to extraction. The frozen samples were pulverized using a bead shocker (Yasui Kikai, Osaka, Japan). Total RNA was extracted using an RNeasy Plant Mini Kit (Qiagen, Hilden, Germany). First-strand cDNA synthesis ensued from 4.5 μl of total RNA using ReverTra Ace reverse transcriptase (Toyobo) with an Oligo(dT)15 primer. qRT-PCR was performed using the GRH6RT1 primer, which were designed potentially to amplify from the T65 allele at *Os04g0165900* and both alleles at *Os04g0166000* (*GRH6*). Rice Ub-CEP52-1 (*Os03g0234200*), which encodes the ubiquitin-fused 60S ribosomal protein L40-1, was amplified using ubiquitin primers as an internal control for qRT-PCR on a MX3000P real-time PCR system (Agilent Technologies, Santa Clara, CA, USA) using the KAPA SYBR FAST qPCR Master Mix Universal Kit (Kapa Biosystems, MA, USA). All the primers used are listed in Supplemental Table 2. The expression level in the samples was quantified relative to the 0-day post-infestation T65 sample.

### Honeydew assay for the estimation of GRH feeding ability

The feeding ability of GRH on the T65 and *GRH6-WK56* NIL was explored using the honeydew test. The Whatman No. 1 filter paper was immersed in a bromocresol green solution (2 mg/ml in ethanol) for 5 min and left to air-dry for an hour. A plastic dish was positioned beneath the seedlings with bromocresol green-treated filter paper being placed on the dish. An overturned plastic cup was then placed on the filter paper. Ten adult female GRH insects, starved for 2 hours, were introduced into each cup and allowed to feed for 24 hours. The alkaline pH of the phloem sap caused the honeydew of the insects to form blue-colored spots from the reaction with bromocresol green on the filter paper (Bharathi *et al.* 2018). The honeydew area was analyzed using ImageJ software.

## Results

### Resistance in Myanmar rice landraces to GRH

**Fig. 1.**
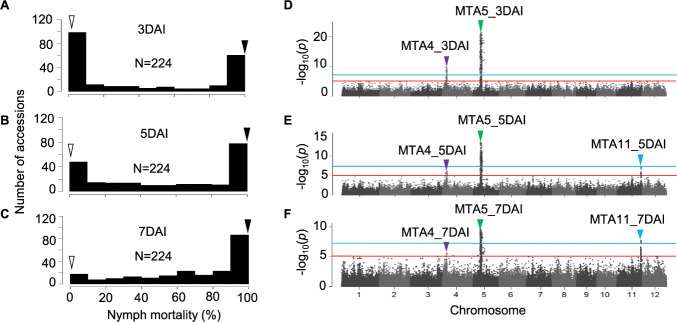
Genome-wide association study for GRH resistance in MIDP (n = 224). (A–C) Frequency distribution of nymph mortality of GRH at 3 days after infestation (DAI) (A), 5DAI (B) and 7DAI (C). White and black arrowheads represent phenotypic scores of T65 and DV85 as susceptible and resistant controls, respectively. (D–F) Manhattan plots for genome-wide associations of GRH resistance at 3DAI (D), 5DAI (E), and 7DAI (F). The red and blue horizontal lines represent suggestive and significant threshold levels at α = 1e-5 and α = 5e-8, respectively. The purple, green and blue arrowheads represent peak –log_10_(*p*) positions of the marker-trait associations on chromosome 4 (MTA4), chromosome 5 (MTA5), and chromosome 11 (MTA11), respectively.

In this study, 224 accessions from the Myanmar *indica* diversity panel (MIDP) were evaluated for resistance to GRH. Nymph mortality was assessed at 3 days after infestation (DAI), 5DAI, and 7DAI. The susceptible control, T65, displayed low GRH nymph mortality (NM) of 1.1% at 3DAI, 1.1% at 5DAI, and 3.4% at 7DAI. In contrast, the resistant control DV85 exhibited high nymph mortality rates of 99.5% at 3DAI, 100% at 5DAI, and 100% at 7DAI (Fig. 1A–1C, Supplemental Table 3). Continuous phenotypic variations in nymph mortality, ranging from 0 to 100%, were observed across the three evaluation times. Accessions showing MR and HR phenotypes increased from 3DAI to 7DAI (Fig. 1A–1C, Supplemental Table 3). At 3DAI, 76, 29, and 119 accessions belonged to the HR, MR, and S classes, respectively. However, the 83 accessions classified as the S class at 3DAI exhibited moderate to high resistance at 5DAI and 7DAI. Of the 224 accessions, 36 exhibited S, 61 displayed MR, and 127 demonstrated HR at 7DAI (Fig. 1A–1C, Supplemental Table 3).

### Genome-wide association study of GRH resistance in MIDP

GWAS using GBLUP for Myanmar *indica* rice landraces was conducted to identify the genomic regions significantly associated with the resistance to GRH. The MIDP diversity panel showed no significant subpopulation structure since the principal component analysis on the MIDP population showed that the first two principal components (PC1 and PC2) accounted only for 6.3% and 3.4% of total genotypic variance (Furuta *et al.* 2024). The PC1 and PC2 scores showed weak correlations with the latitudinal and longitudinal origins of the accessions (PC1 score vs. latitude: *r* = 0.39; PC2 score vs. longitude: *r* = 0.22) (Furuta *et al.* 2024). This suggests that although geographical factors might influence genetic variations in Myanmar’s rice landraces, they do not significantly drive the genetic differentiation within the subpopulation. Therefore, the population structure was not included in the model of GBLUP.

**Fig. 2.**
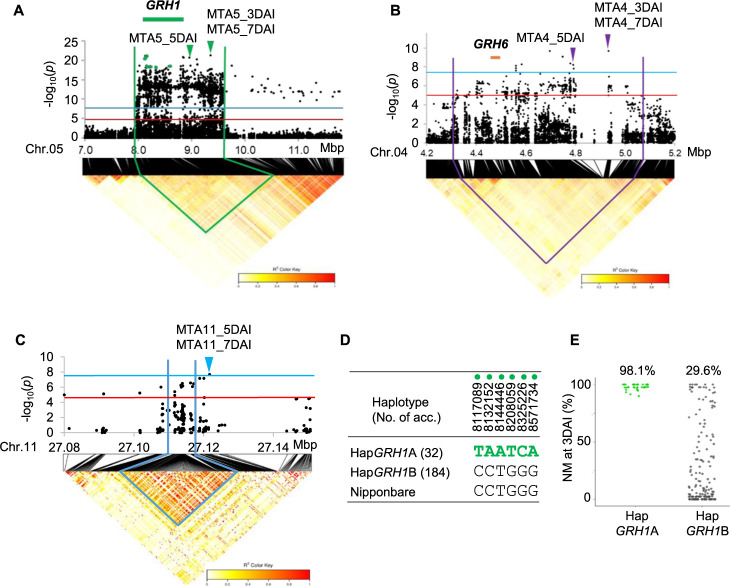
Significant associations with GRH resistance in MIDP (n = 224). (A–C) Local Manhattan plots for MTA5 at 3 days after infestation (DAI) (A), MTA4 at 3DAI (B) and MTA11 at 7DAI (C). The red and blue horizontal lines represent suggestive and significant threshold levels at α = 1e-5 and α = 5e-8, respectively. The green, purple and blue arrowheads indicate highest peaks of MTA5, MTA4 and MTA11 regions respectively. The green and orange bars represent the high resolution mapping region of *GRH1* (Park *et al.* 2013) and *GRH6* (Phi *et al.* 2019). Green dots represent SNP markers used for haplotype analysis for *GRH1*. The lower parts of the panel A, B, C represent LD heat map surrounding MTA5, MTA4 and MTA11 respectively. Green, purple and blue lines represent the LD regions harboring MTA5, MTA4 and MTA11 respectively. (D) Haplotype variations at *GRH1*. (E) Nymph mortality (NM) of GRH by the haplotype variations at *GRH1*. The number on the scatter plot is mean value of the nymph mortality of each haplotype.

On chromosome 5, the marker-trait associations MTA5_3DAI, MTA5_5DAI, MTA5_7DAI were detected with highest –log_10_(*p*) values of 21.3, 13.4, and 9.5, respectively (Fig. 1D–1F, Table 1) within the apparent LD block spanning from 7.97 to 9.76 Mbp of the Nipponbare reference genome in the panel (Fig. 2A). Since this LD block contained the previously reported resistance gene, *GRH1* (Kadowaki *et al.* 2003, Tamura *et al.* 1999), which was delimited within a 670 kb region (Park *et al.* 2013), it was speculated that MTA5 corresponds to *GRH1* and predominantly control the resistance to GRH in MIDP (Fig. 2A).

On chromosome 4, the marker-trait associations: MTA4_3DAI, MTA4_5DAI and MTA4_7DAI were detected with –log_10_(*p*) values of 9.7, 8.2, and 5.6, respectively (Fig. 1D–1F, Table 1). The genomic region with SNP markers having –log_10_(*p*) values above suggestive threshold lines spanned between 4.3 and 5.1 Mbp regions of the Nipponbare reference genome in the panel (Fig. 2B). The previously reported gene, *GRH6* (Fujita *et al.* 2004, Tamura *et al.* 2004) delimited within 31.2 kb of the genomic region between the DNA markers c60k and 7L16f, corresponding to 4,460,301–4,491,541 bp in the reference sequence of Nipponbare (Phi *et al.* 2019) was located in this genomic region. Although the candidate region of *GRH6* and the peak SNPs of MTA4 at 3DAI, 5DAI, and 7DAI were included in the weak LD block, MTA4_3DAI and MTA4_7DAI were approximately 400kb far from *GRH6* (Fig. 2B). Therefore, it remained need to confirm whether *GRH6* was candidate gene for this genomic region or not.

On chromosome 11, the marker-trait associations on chromosome 11 (MTA11_5DAI and MTA11_7DAI) were detected at the later stages of infestation at 5DAI and 7DAI with –log_10_(*p*) values of 7.1 and 7.7 respectively (Fig. 1E, 1F, Table 1). The LD block located closely to MTA11_5DAI and MTA11_7DAI ranged from 27.11 to 27.12 Mbp of the Nipponbare reference genome in the panel (Fig. 2C). No previously identified genes or QTLs for GRH resistance were found within this region, suggesting that MTA11 was likely to be a novel QTL for GRH resistance. Quantile-quantile (QQ) plots of –log_10_(*p*) at 3DAI, 5DAI, and 7DAI show inflated –log_10_(*p*) values from the expected distribution under the null hypothesis, where there are no QTL. However, as the SNPs with inflated –log_10_(*p*) values were derived from the MTA4, MTA5, and MTA11 regions, these high –log_10_(*p*) values were considered to be derived from significant QTLs (Supplemental Fig. 1).

### GWAS of GRH resistance in MIDP after excluding the predominance effect of *GRH1*

To examine more precise locations of the detected MTAs on chromosomes 4 and 11, we defined haplotypes at the most predominantly affected locus *GRH1* for the GRH resistance in MIDP, and GWAS was performed again using the MIDP accessions without the accessions carrying the resistance haplotype at *GRH1*.

Haplotype analysis at the *GRH1* region were performed using the six SNPs from the highest –log_10_(*p*) values in the candidate region of fine-mapping of *GRH1* at 8,117,089, 8,132,152, 8,144,446, 8,208,059, 8,325,226, and 8,571,734 bp (Fig. 2A, 2D). The analysis showed that the two genomic haplotypes, Hap*GRH1*A and Hap*GRH1*B, were found associated with resistant and susceptible nymph mortality, respectively (Fig. 2D, 2E, Supplemental Table 4). Thirty-two accessions possessing the Hap*GRH1*A haplotype showed significantly higher nymph mortality at 3DAI, whereas accessions carrying the haplotype Hap*GRH1*B showed a wide range of variation in nymph mortality (Fig. 2E, Supplemental Table 4).

**Fig. 3.**
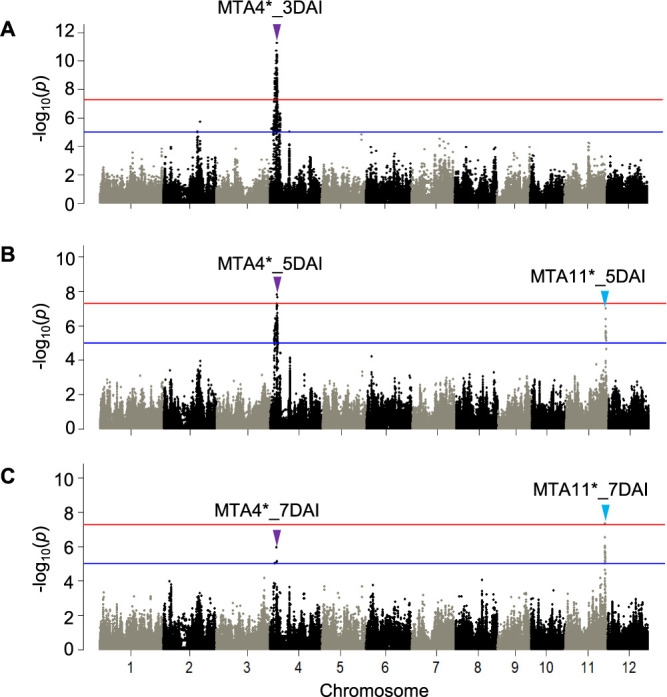
Manhattan plots for genome-wide associations of nymph mortality (%) of green rice leafhopper infested to the accessions of MIDP (n = 184) using the accessions carrying the susceptible haplotype at *GRH1*, Hap*GRH1*B at 3 days after infestation (DAI) (A), 5DAI (B) and 7DAI (C). The blue and red horizontal lines represent suggestive and significant threshold levels at α = 1e-5 and α = 5e-8, respectively indicating the possible locus conferring resistance to GRH. The purple and blue arrowheads represent peak –log_10_(*p*) positions at the marker-trait associations on chromosome 4 (MTA4) and chromosome 11 (MTA11), respectively.

**Fig. 4.**
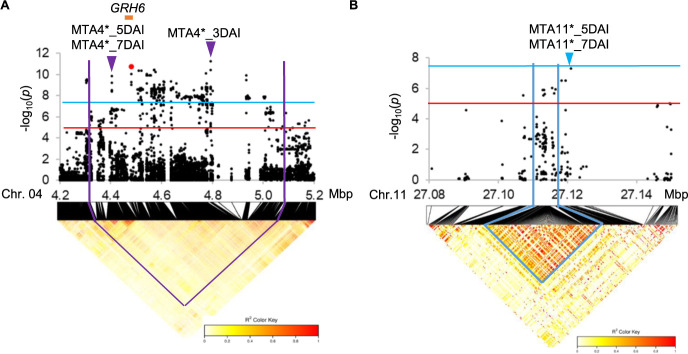
Significant associations with GRH resistance in MIDP (n = 184) using the accessions carrying the susceptible haplotype at *GRH1*, Hap*GRH1*B. (A, B) Local Manhattan plots for MTA4 at 3 days after infestation (DAI) (A), MTA11 at 7DAI (B). The red and blue horizontal lines represent suggestive and significant threshold levels at α = 1e-5 and α = 5e-8, respectively. The purple and blue arrowheads indicate highest peaks of MTA4, MTA11 regions respectively. The red dot represents the SNP with second highest peak of MTA4 region. The orange bars represent the high resolution mapping region of *GRH6* (Phi *et al.* 2019). The lower parts of the panel A, B represent LD heat map surrounding MTA4 and MTA11 respectively. Purple and blue lines represent the LD regions harboring MTA4 and MTA11 respectively.

Next, the GWAS using the 184 accessions carrying the susceptible haplotype at *GRH1*, Hap*GRH1*B, was conducted. The marker-trait associations on chromosome 4, MTA4*_3DAI, MTA4*_5DAI, and MTA4*_7DAI, were detected at 4,791,920 bp and 4,404,524 bp with the –log_10_(*p*) values of 11.2, 7.8 and 5.9 respectively (Fig. 3, Table 2). The second highest peak SNP detected at 3DAI was located at 4,481,803 bp within the high-resolution mapping region of *GRH6* (Fig. 4A). Other significantly associated SNPs at 5DAI and 7DAI were observed around the high-resolution mapping region of *GRH6* (Fig. 4A). These results suggested that removing the resistant accessions harboring the Hap*GRH1*A allow to more accurately finding marker-trait associations around the *GRH6* region.

Similarly, MTA11*_5DAI and MTA11*_7DAI were identified at 27,121,054 bp with the –log_10_(*p*) values of 7.0 and 7.3 respectively (Fig. 3B, 3C, Table 2). These positions were similar with the result of previous GWAS (Fig. 4B).

### Gene cloning of *GRH6*

**Fig. 5.**
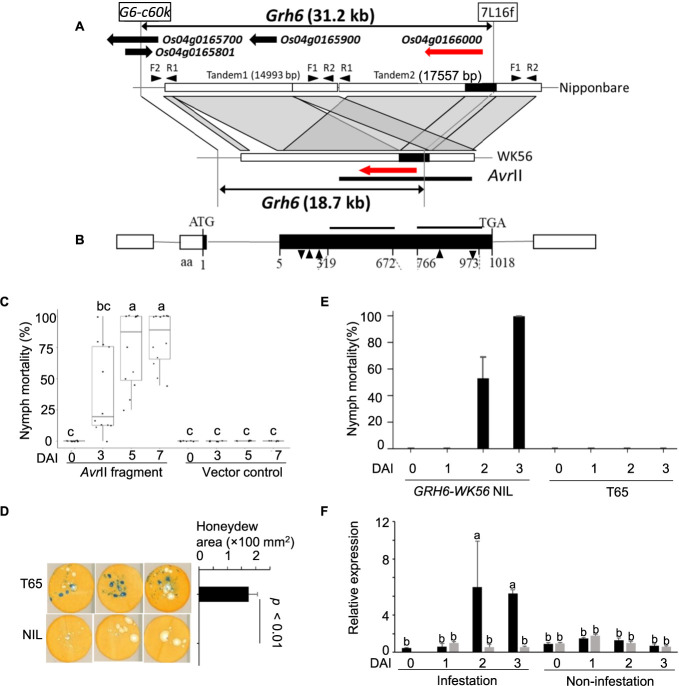
Map-based cloning of *GRH6*. (A) Genetic and physical position of *GRH6*. G6-c60k and 7L16f represent the flanking markers used in delimitation of *GRH6* region (Phi *et al.* 2019). The predicted genes annotated in the Rice Genome Annotation Project Database (RAP-DB) were shown as black and red arrows. The red arrow represents *Os04g0166000* encoding LRR protein and located on the tandemly duplicated region 2 (tandem2) in Nipponbare sequence. Black box on tandem2 represents the genomic segment lacked in the tandemly duplicated region 1 (tandem1). The black bar represents the *Avr*II restricted fragment containing *Os04g0166000* derived from *O. nivara* bacterial artificial chromosome (BAC) clone IRGC105715-26E15. Primers for confirmation of the tandem1 and tandem2 were shown in arrowheads. (B) Structure of *GRH6-WK56* (*Os04g0166000*). White boxes represent the 5ʹ and 3ʹ untranslated regions (UTRs), and black boxes represent the coding sequence. The black bars represent the deduced LRR domains. Black downward and upward arrowheads represent insertion and deletion of nucleotide respectively. (C) Nymph mortality of GRH in T_0_ transgenic plants carrying *Avr*II fragment and vector control. (D) Honeydew assay for GRH after 24-h feeding on *GRH6-WK56* NIL and T65. Blue-rimmed spots were evaluated as areas of phloem-derived honeydew. (E) Nymph mortality of GRH in *GRH6-WK56* NIL and T65. (F) Time course expression of *Os04g0166000* in the leaf blades of infested and non-infested of *GRH6-WK56* NIL and T65 using three replicates. The black and grey bars represent *GRH6-WK56* NIL and T65 respectively. Zero-day after infestation (0DAI) is the time point before GRH infestation. Relative expression to the samples at 0DAI in T65 was shown. Bars with the same letter are not significantly different at *P* < 0.05 by Tukey-Kramer test.

*GRH6* had been mapped on the short arm of chromosome 4 in the Surinam cultivar “SML17” (Tamura *et al.* 2004) and in a wild accession of *O. nivara*, IRGC105715 (WK56) (Fujita *et al.* 2004). In *O. nivara*, *GRH6* region had been delimited to 32.2 kbp size of the Nipponbare genome (4,460,301 to 4,491,541 bp) between the markers G6-c60k and 7L16f (Phi *et al.* 2019). However, the causal gene of *GRH6* had not been detected yet and the molecular mechanism underlying this gene is still unknown. Therefore, the causal locus of *GRH6* was identified by map-based cloning using the *O. nivara* bacterial artificial chromosome (BAC) clone IRGC105715-26E15 in this study. The genomic region of *GRH6* contained four predicted genes putatively encoding a cysteine synthase (*Os04g0165700*), a hypothetical protein (*Os04g0165801*), and two conserved hypothetical proteins containing a leucine-rich repeat (LRR) domain (*Os04g0165900* and *Os04g0166000*) (Phi *et al.* 2019, Fig. 5A). The predicted genes *Os04g0165900* and *Os04g0166000* were located in the tandemly duplicated genomic regions, tandem1 (14,993 bp) and tandem2 (17,557 bp), in the reference sequence of Nipponbare (Fig. 5A). The tandem duplication was also confirmed in T65 (Supplemental Fig. 2). Sequencing of the *O. nivara* bacterial artificial chromosome (BAC) clone IRGC105715_26E15 and PCR amplification from *GRH6-WK56* NIL revealed that the tandem1 genomic region was deleted, and only one copy of the gene (*Os04g0166000*) (Fig. 5A, Supplemental Fig. 2). The genomic sequence is deposited in the DNA Data Bank of Japan (Accession No. LC781942). A total of 12.6 kbp genomic DNA fragment containing *Os04g0166000* and 4.7 kb length of 5ʹ region of the gene was restricted from *O. nivara* bacterial artificial chromosome (BAC) clone IRGC105715-26E15 with the restriction enzyme *Avr*II and inserted into the Ti plasmid binary vector pPZP2H-*lac* (Fuse *et al.* 2001) which was digested with *Xba*I. The nymph mortality of T_0_ plants containing the *Avr*II fragment gradually increased, and all T_0_ plants showed a moderate to high resistance to GRH at 7DAI (Fig. 5C), whereas T_0_ plants harboring the empty vector showed susceptible to GRH. These results demonstrated that the WK56 allele at *Os04g0166000*, which encodes the LRR protein, is responsible for GRH resistance and designated as *GRH6-WK56*. Using the full-length Nipponbare cDNA, the deduced coding sequence of *GRH6-WK56* was 1,018 aa in length, encoding two LRR domains (Fig. 5B). *Os04g0165900*, on the tandem1 segment of Nipponbare lacked the N-terminal region, first codon, transcription initiation site, and promoter region (Fig. 5A, black box). In contrast, the other *GRH6-WK56* homolog, CDS at *Os04g0166000*, had two insertions, three deletions, and 65 amino acid substitutions in Nipponbare (Fig. 5B, Supplemental Fig. 3).

The feeding ability of GRH on *GRH6-WK56* NIL and T65 was investigated using honeydew deposit from ten adult female GRH. A large blue-rimmed spot area was observed in T65 due to honeydew deposits derived from the phloem 24 hours after infestation (Fig. 5D). However, no blue spots were observed in *GRH6-WK56* NIL, suggesting that *GRH6* confers resistance to GRH by inhibiting its sucking from the phloem. The nymph mortality of GRH significantly increased at 2DAI and 3DAI in the *GRH6-WK56* NIL (Fig. 5E). In contrast, GRH nymph on T65 showed no mortality, suggesting that *GRH6* sufficiently induced nymph mortality at 2DAI and 3DAI and lead to insect death (Fig. 5E).

qRT-PCR was conducted using the primers which were designed potentially to amplify from the T65 allele at *Os04g0165900* and both alleles at *Os04g0166000* (*GRH6*). The expression level of *Os04g0166000* on the leaf blade of the GRH infested *GRH6-WK56* NIL at 1DAI was unchanged to 0DAI but was significantly up-regulated at 2DAI and 3DAI. But the expression level of *Os04g0165900* and *Os04g0166000* was unchanged in infested T65 leaf. In contrast, the expression was not up-regulated in non-infested leaves of both *GRH6-WK56* NIL and T65. These results demonstrated that the expression of only *GRH6-WK56* was up-regulated by GRH infestation (Fig. 5F). Since the high-resolution mapping of *GRH6* revealed that the insertion-deletion polymorphism marker, 7L16f, delimited the candidate region of *GRH6* in its downstream region (Phi *et al.* 2019), it was postulated that 16 of single nucleotide or insertion-deletion polymorphisms within the promoter region of *GRH6* between the T65 and WK56 might determine the transcriptional differences between the two alleles at *GRH6* (Supplemental Fig. 4).

### Haplotype estimation for causal gene of *GRH6* in MIDP

**Fig. 6.**
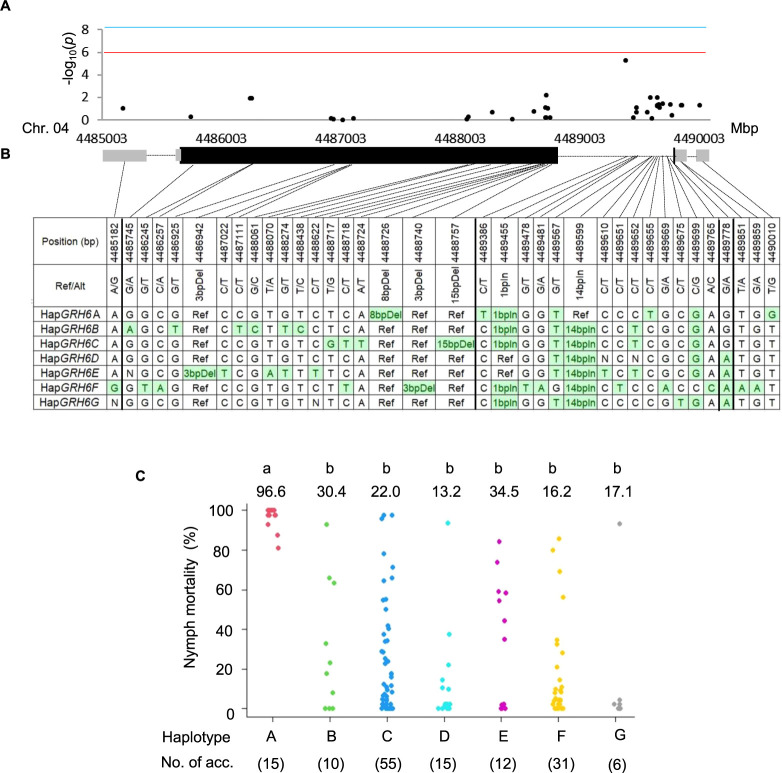
Haplotype estimation for causal gene of *GRH6* (*Os04g0166000*) in MIDP. (A) Associations between SNPs, InDel markers and nymph mortality of GRH at *Os04g0166000*. (B) Gene structure of *GRH6* and haplotype variations in MIDP at *Os04g0166000* based on 15 SNPs and 4 InDels in coding sequence (CDS), 4 SNPs in untranslated region (UTR), 12 SNPs and 2 InDels in intron. Black and grey boxes represent the exon and UTR region, and black line represents the intron. Haplotypes with frequencies exceeding 0.05 were considered major haplotype variants. (C) Nymph motility (%) of GRH in MIDP containing in seven major haplotypes of *Os04g0166000* at 3 days after infestation. The number on the scatter plot is mean value of the nymph mortality (%) of each haplotype. Different alphabets indicate that mean values of each haplotype was significantly different each other by Tukey-Kramer test.

Genetic effects of the haplotypes at the causal gene of *GRH6* (*Os04g0166000*) was investigated using the 15 SNPs and 4 InDels in coding sequence region (CDS), 4 SNPs in untranslated region, 12 SNPs and 2 InDels in intron region (Fig. 6A, 6B) in the MIDP accessions not carrying the resistance haplotype Hap*GRH1*A at 3DAI. Genetic effect at MTA11 to nymph mortality was almost negligible at 3DAI (Fig. 3A). Of the identified seven major haplotypes (Hap*GRH6*A, Hap*GRH6*B, Hap*GRH6*C, Hap*GRH6*D, Hap*GRH6*E, Hap*GRH6*F, Hap*GRH6*G), all the accessions in Hap*GRH6*A showed highly resistance to GRH at 3DAI whereas the average nymph mortality of the other haplotypes (Hap*GRH6*B-G) showed susceptible to GRH with less than 35% nymph mortality without significant difference among them (Fig. 6B, 6C). These results suggested that Hap*GRH6*A correspond to GRH resistance. A wide range of nymph mortality (%) of GRH from 0 to 100% was observed in the other six haplotypes (Fig. 6B, 6C), suggesting that the phenotypic variation might be due to background QTL. However, the resistance allele donor at *GRH6* of wild species, WK56, and T65 did not possess the resistance haplotype (*GRH6*HapA) (Data not shown). Expanding haplotype estimation constructed in Myanmar cultivated rice to the wild rice may not be straightforward.

### Validation of MTA11 in bi-parental segregating population

**Fig. 7.**
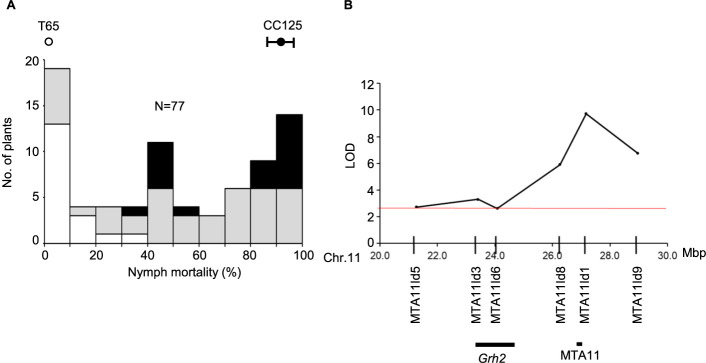
Identification of *qGRH11* in F_2_ population derived from the cross between CC125, resistant accession of MIDP, and susceptible cultivar T65. (A) Frequency distribution of nymph mortality of GRH at 7 days after infestation. Black, grey and white boxes represent the homozygous for CC125, heterozygous, and homozygous for T65 plants at the MTA11Id1 InDel marker, respectively. (B) Logarithm of odds (LOD) score in marker regression using six InDel markers. The red horizontal line represents 1% significant level in 1000 times permutation test.

To validate MTA11 as a QTL, QTL analysis was conducted in the bi-parental F_2_ population derived from the cross between CC125, the Myanmar accession carrying the resistance haplotype at MTA11 (HapMTA11A) and the susceptible *japonica* cultivar T65 using the six InDel markers around MTA11 region. F_2_ individuals showed continuous segregation in nymph mortality of GRH (Fig. 7A). A significant QTL, *qGRH11*, was found on chromosome 11 with the peak LOD score of 9.7, explaining 43.7% of the phenotypic variation (Table 3). The QTL peak was represented by the InDel marker MTA11Id1 at 27,177,381 bp around the MTA11 region, and QTL region covered from MTA11Id8 (26,265,353 bp) to MTA11Id9 (28,967,652 bp) overlapping with the MTA11 region (27,107,110 bp to 27,121,054 bp) (Fig. 7B). While the previously reported gene, *GRH2*, was located near the *qGRH11* region, the LOD value of *GRH2* region was lower than that of *qGRH11* speculating that the QTL closely linked to MTA11 was likely a novel GRH resistance locus on chromosome 11 (Fig. 7B).

### Haplotype analysis for GRH resistance in MIDP at MTA11

**Fig. 8.**
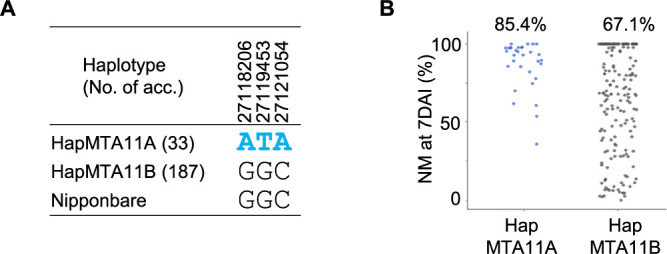
Haplotype estimation for GRH resistance in MIDP at MTA11. (A) Haplotype variations estimated by the three strongest associated SNPs with highest –log_10_(*p*) values within the genomic region of MTA11. (B) Nymph mortality (NM) of GRH by the haplotype variations at MTA11. The number on the scatter plot is mean value of the nymph mortality of each haplotype.

Two major genomic haplotypes (HapMTA11A and HapMTA11B), estimated by the three strongest associated SNPs at 27,118,206, 27,119,453 and 27,121,054 bp with highest –log_10_(*p*) values within the genomic region of MTA11 were observed (Fig. 8A). Thirty-three accessions harboring the HapMTA11A haplotype exhibited moderate to high nymph mortality at 7DAI (Fig. 8B) but not at 3DAI and 5DAI (Supplemental Table 4), demonstrating that HapMTA11A contributed to GRH resistance with a delayed resistance effect, whereas the accessions harboring the haplotype HapMTA11B showed high variation in nymph mortality (Fig. 8B).

### Distribution of combined resistant haplotypes in Myanmar landraces

**Fig. 9.**
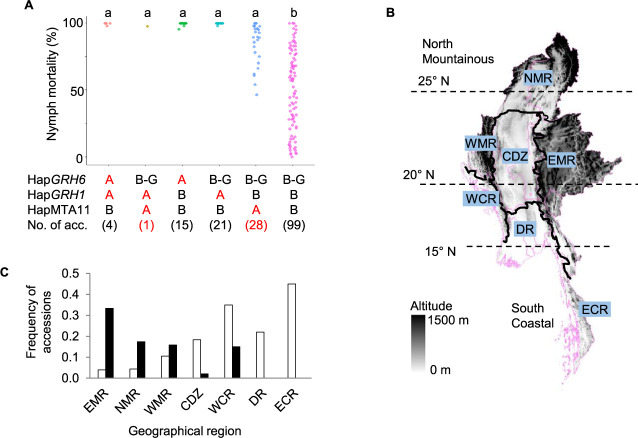
Combination and distribution of resistant haplotypes in MIDP (n = 168). (A) Nymph mortality of GRH at 7 days after infestation by the resistant haplotype combinations. The number in the parenthesis represents the number of accessions in each haplotype combination. Red letter A represents the resistance haplotype and the black letter B–G represents the susceptible haplotype at *GRH1*, *GRH6* and MTA11. (B) The seven geographical regions of Myanmar. NMR, EMR, WMR, CDZ, WCR, DR, and ECR represent the northern mountainous region, eastern mountainous region, western mountainous region, central dry zone, western coastal region, delta region, and eastern coastal region, respectively. The color changing from grey to black represent low to high altitude. (C) Geographical distribution of resistance haplotypes in MIDP. White bars represent the frequency of accessions carrying Hap*GRH6*A or Hap*GRH1*A or both of them. Black bar indicates the frequency of accessions carrying HapMTA11A.

Combinations of resistance haplotypes in MIDP were also investigated using 168 accessions after excluding the accessions with unknown haplotypes (Fig. 9A). No accessions carrying of the three resistance haplotypes were identified. Forty-one accessions that carried at least one or more resistance haplotypes at major resistance genes including *GRH1* and *GRH6* (Hap*GRH6*A/Hap*GRH1*A/HapMTA11B, Hap*GRH6*B-G/Hap*GRH1*A/HapMTA11A, Hap*GRH6*A/Hap*GRH1*B/HapMTA11B, Hap*GRH6*B-G/Hap*GRH1*A/HapMTA11B) exhibited high levels of resistance (Fig. 9A, Supplemental Table 4). Twenty-eight accessions belonging to the resistance haplotype at MTA11 (Hap*GRH6*B-G/Hap*GRH1*B/HapMTA11A) exhibited low to high nymph mortality at 3DAI and showed moderate to high nymph mortality at 7DAI (Fig. 9A, Supplemental Table 4) assuming that this is likely to be explained by the delay resistance effect of HapMTA11A. Nevertheless, the remaining accessions carrying no resistance haplotype at the loci detected in this study (Hap*GRH6*B-G/Hap*GRH1*B/HapMTA11B) showed a wide phenotypic variation in nymph mortality ranging from 0 to 100% (Fig. 9A, Supplemental Table 4). This suggests that the resistance may be due to other unidentified QTLs in this study. Cultivars with unknown alleles of GRH resistance are potential donors for exploring novel alleles that confer GRH resistance.

The geographical distribution of the combination of resistant haplotypes was further investigated. Accessions were initially collected from seven geographical regions: the northern mountainous region (NMR), eastern mountainous region (EMR), western mountainous region (WMR), central dry zone (CDZ), western coastal region (WCR), delta region (DR), and eastern coastal region (ECR) (Fig. 9B). Accessions harboring resistant haplotypes at major resistance genes including *GRH6* and *GRH1* (Hap*GRH6*A and Hap*GRH1*A) were mainly observed in the CDZ and southern region of Myanmar including WCR, DR, and ECR, and their frequencies were higher than those in mountainous regions, including NMR, EMR, WMR (Fig. 9B, 9C, Supplemental Table 5). In contrast, accessions carrying resistance haplotype at MTA11 (HapMTA11A) was mainly distributed in EMR and partly in NMR, WMR and WCR (Fig. 9B, 9C, Supplemental Table 5) speculating that MTA11 is likely the main resistance QTL or gene for accessions from mountainous regions.

## Discussion

### QTL governing delayed resistant effect

In the present study, we identified 188 accessions with MR and HR at 7DAI in the panel. Among the 188 accessions, 105 reached MR and HR levels within 3DAI. Conversely, the subsequent 83 accessions showed delayed expression of resistance, achieving MR to HR levels at 5DAI or 7DAI (Fig. 1A–1C, Supplemental Table 3). GWAS detected three genomic regions conferring resistance to GRH on chromosomes 4 (MTA4), 5 (MTA5), and 11 (MTA11). MTA4 and MTA5 were identified in all evaluation periods with high –log_10_(*p*) values. However, MTA11 was found at 5DAI and 7DAI with lower –log_10_(*p*) values, suggesting that MTA4 and MTA5 were major resistance genes, and that MTA11 may be a major QTL with delayed resistance (Fig. 1D–1F, Table 1).

### Identity of MTA with previously reported loci

On chromosome 5, *GRH1* corresponded to resistance in this panel, as *GRH1* and MTA5_3DAI, MTA5_5DAI and MTA5_7DAI were present within the same apparent LD block (Fig. 2A). The peak SNPs of MTA5 region detected at different evaluated periods: MTA5_3DAI, MTA5_5DAI and MTA5_7DAI were not stable in position even though these SNPs belonged in the same LD block containing *GRH1* (Fig. 2A). It is possible that suggested positions of QTL may shift between different time points or trials because of the high phenotypic variance and dynamic nature of plant-insect interaction. Similar trend was observed between *GRH6* and peak SNPs of MTA4: MTA4_3DAI, MTA4_5DAI and MTA4_7DAI on chromosome 4 (Fig. 2C).

One of the previously reported genes, *GRH2* was found on the long arm of chromosome 11 between the makers RM5349 and G2-10 (23.3 to 24.7 Mbp) (Kadowaki *et al.* 2003). In *GRH2*-NIL, 75% nymph mortality was observed at 3DAI (Fujita *et al.* 2010) suggesting *GRH2* expressed resistance at early stage. However, MTA11 was detected at later stage in GWAS (5DAI and 7DAI) (Fig. 1E, 1F), and located in a different region approximately 2.4 Mbp far from *GRH2* speculating that MTA11 is likely a novel locus independently from *GRH2*, and it was validated using bi-parental F_2_ population (Fig. 7B, Table 3). However, the difference between the regions of *GRH2* and *qGRH11* was difficult to discriminate due to limited number of InDel markers and low resolution mapping population (F_2_). Therefore, identification of candidate region for MTA11 region need to be further confirmed using high resolution mapping populations such as CSSL and RILs.

On chromosome 4, the highest peak SNP of MTA4 region at 4,932,132 bp (MTA4_3DAI) and the other high significantly associated SNPs were far from *GRH6*, and they were located within the weak LD block (Fig. 2B). After excluding the predominance effect of major resistance gene in this population, *GRH1*, marker-trait associations around *GRH6* region were more accurately observed, suggesting that *GRH6* contributed to GRH resistance in this diversity panel (Fig. 4A).

### Haplotype estimation

GWAS identified three genomic regions including *GRH1*, *GRH6* and MTA11 associated with GRH resistance in MIDP. Using the highest associated SNPs within the genomic region of *GRH1* and MTA11, we estimated the accessions carrying resistance and susceptible haplotype (Supplemental Table 4), and all the accessions carrying the resistant haplotype at *GRH1* (Hap*GRH1*A) and MTA11 (HapMTA11A) exhibited moderate to high resistance to GRH (Figs. 2E, 8A, Supplemental Table 4). The haplotype analysis performed for the causal gene of *GRH6* which was identified by gene cloning in this study revealed that Hap*GRH6*A correspond to GRH resistance in this panel (Fig. 6C). However, some functional SNPs and InDels within the *GRH6* gene region seemed to be excluded after filtering with maximum missing frequency of less than 0.1 and minor allele frequency more than 0.05. As *GRH6* is located in the tandemly duplicate region on chromosome 4, the functional SNPs at *GRH6* may not be detected well due to incorrect or missed SNP calling resulted from the complexity of short read mapping on the duplicate genomic region (Ko *et al.* 2022).

Analysis of haplotype combinations showed that 69 accessions possessed at least one resistant haplotype at the detected loci and were resistant to GRH (Fig. 9A, Supplemental Table 4). Conversely, 71 accessions lacking a resistant haplotype at any detected locus still demonstrated moderate-to-high resistance to GRH (Fig. 9A, Supplemental Table 4). This implies that these accessions are invaluable donors of novel QTLs that confer GRH resistance. GWAS and haplotype analysis accurately identified three known genes and three novel genes significantly linked to the heading date, and uncovered functional and nonfunctional alleles of each detected gene in North Korean rice populations (Jadamba *et al.* 2022). These findings suggest that haplotype analysis can identify genomic regions related to traits more accurately than a single SNP marker.

Accessions carrying one or more major resistance haplotypes at the major reported genes: *GRH6* and *GRH1* (Hap*GRH6*A and Hap*GRH1*A) were mainly distributed in the southern area of Myanmar, including WCR, DR and ECR (Fig. 9B, 9C, Supplemental Table 5). Population structure analysis of the MIDP showed that while geographical factors might influence genetic variations in Myanmar’s rice landraces, they do not significantly drive the genetic differentiation within the subpopulation (Furuta *et al.* 2024). GRH is an endemic species distributed in East Asia and not in Myanmar. Therefore, the resistant haplotype distributions at the GRH resistance locus are likely to be resulted from the genetic drift from the ancestral populations or hitchhiking by selection to the other traits without intentionality. Another possibility is that constant exposure of host plants to insect pests was occurred in southern Myanmar because of year-round intensive rice growing, and a warm and humid climate favorable for the proliferation of insect pests in these areas. Such kind of condition presumably enrichment to the major resistance genes (*GRH6* and *GRH1*) in southern regions. While GRH was not found in Myanmar, one of the related species, green leafhopper: GLH (*Nephotettix virescens*) was mainly distributed in South East Asia including Myanmar (Wilson and Claridge 1985). Some GRH resistance genes also resistance to GLH, for example, *GRH2* and *GRH4* in DV85 rice cultivar (Yasui and Yoshimura 1999). Since *GRH6* was found within the region of *Glh14*, we speculated that *GRH6* locus might have undergone indirect selection to GLH resistance in Southern parts of Myanmar. Same phenomenon was likely occurred in indirect selection for BPH resistance since *GRH6* located at the same position of *BPH40* (Shi *et al.* 2021). The current resistant haplotype distribution is likely to resulted from the selection of resistance to BPH and GLH.

### Genetic differences between resistant and susceptible alleles

Expression analysis revealed that the expression of *GRH6-WK56* NIL was induced by GRH infection (Fig. 5F). Transformation of *Avr*II fragment induced GRH resistance to the T65, suggesting that a reduced 5ʹ region (promoter region) of *GRH6* in WK56 might control the expression and induced GRH resistance. The gene promoter region plays a critical role in controlling gene expression by binding transcription factors to various *cis*-regulatory elements in the promoter (Chang *et al.* 2023). In this study, insertions, deletions, and nucleotide polymorphisms were found in the promoter region of *GRH6* between WK56 and T65 (Supplemental Fig. 4), suggesting that polymorphisms in this promoter region enhance gene expression in *GRH6-WK56* NIL. Therefore, we hypothesized that the promoter region of *GRH6* in WK56 might contain specific sequences or motifs recognized by transcription factors activated in response to insect infestation, leading to increased gene expression in *GRH6-WK56* NIL. Additionally, insertion, deletion and polymorphism of amino acid in the coding region of *GRH6* especially the LRR domains, the recognition site for effector, were observed between WK56 and Nipponbare (Supplemental Fig. 3), assuming that amino acid polymorphism may affect the structure and function of the recognition of effector. Further experiments including evaluation of over expressor of the susceptible Nipponbare or T65 allele at *GRH6* or swapping promoter and CDS between the resistant and susceptible alleles play an important role to identify the functional polymorphism between the alleles.

### Function of *GRH6*

*GRH6-WK56* encodes the protein with two LRR domains (Fig. 5B). In rice, most cloned R genes encode CC-NBS-LRR domain-containing proteins, for example (*BPH14*, *BPH26*, *BPH9*, *BPH18*, *BPH30*) (Du *et al.* 2009, Ji *et al.* 2016, Shi *et al.* 2021, Tamura *et al.* 2014, Zhao *et al.* 2016). NBS-LRR proteins recognize pathogen effectors when the pathogens secrete molecules (effectors) into plant cells to suppress the immune response of the plant and trigger potent innate immune responses (McHale *et al.* 2006). *BPH9*, which encodes CC-NBS-NBS-LRR, activates salicylic acid and jasmonic acid signaling pathways in rice plants upon BPH feeding, and confers resistance to BPH (Zhao *et al.* 2016). *BPH14* encoding CC-NBS-LRR induced callose deposition in sieve tubes, reducing BPH feeding, development, and survival (Du *et al.* 2009). Both *BPH30* (allelic to *Os04g0115650*) and its homolog *BPH40* (allelic to *Os04g0166000*) encode the protein with two LRR domains enhanced cellulose and hemicellulose synthesis, making the cell walls stiffer and sclerenchyma thicker; these were the physical barriers preventing BPH from feeding on the phloem (Shi *et al.* 2021). Since *GRH6* is the same locus as *BPH40*, there is a possibility that the function of *GRH6* was likely to be similar that of *BPH40* which is related to cell wall fortification in the sclerenchyma and inhibition of the feeding of insects to the phloem. The honeydew assay revealed *GRH6* prevent the feeding of GRH to the phloem in *GRH6-WK56* NIL. The detailed molecular functions of *GRH6* should be investigated in future studies.

In this study, we estimated the resistant haplotypes associated with GRH resistance in Myanmar *indica* landraces using genome-wide association mapping. These resistant donors and their genetic information could be valuable for developing locally-adapted GRH resistant rice cultivars.

## Author Contribution Statement

DF, HY, and YY conceptualized this study. The overall research was conducted by NMK. DF and HY provided advice on the experiments. AY and HY provided seeds. HK, JW, and TM performed the BAC screening and sequencing. The original manuscript was written by NMK, and reviewed by NMK and YY.

## Supplementary Material

Supplemental Figures

Supplemental Tables

## Figures and Tables

**Table 1.** Marker-traits associations conferring resistance to GRH based on genome-wide association study in MIDP (n = 224)

Marker trait association (MTA)	Chr.	Position of MTAs (bp)	−log_10_(*p*)	SNP allele (Allele 1/Allele 2)	NM (%)*^a^*		Reported gene flanking to MTA
					Allele 1 homozygous	Allele 2 homozygous	
MTA4_3DAI	4	4,932,132	9.7	A/G	35.0	97.2	*GRH6*
MTA5_3DAI	5	9,349,253	21.3	G/A	28.3	97.8	*GRH1*
MTA4_5DAI	4	4,791,920	8.2	A/G	50.2	96.1	*GRH6*
MTA5_5DAI	5	8,952,557	13.4	G/T	29.1	98.1	*GRH1*
MTA11_5DAI	11	27,121,054	7.1	C/A	52.5	75.7	
MTA4_7DAI	4	4,932,132	5.6	A/G	66.1	99.7	*GRH6*
MTA5_7DAI	5	9,349,253	9.5	G/A	62.3	99.6	*GRH1*
MTA11_7DAI	11	27,121,054	7.7	C/A	66.0	86.5	

*^a^* NM represent nymph mortality.

**Table 2.** Marker-traits associations conferring resistance to GRH based on genome-wide association study in MIDP using the accessions carrying susceptible haplotype at *GRH1*, Hap*GRH1*B (n = 184)

Marker trait association (MTA)	Chr.	Position of MTAs (bp)	−log_10_(*p*)	SNP allele (Allele 1/Allele 2)	NM (%)*^a^*		Reported gene flanking to MTA
					Allele 1 homozygous	Allele 2 homozygous	
MTA4*_3DAI	4	4,791,920	11.2	A/G	22.4	88.0	*GRH6*
MTA4*_5DAI	4	4,404,524	7.8	G/A	39.8	90.8	*GRH6*
MTA11*_5DAI	11	27,121,054	7.0	C/A	41.5	75.1	
MTA4*_7DAI	4	4,404,524	5.9	G/A	57.4	95.8	*GRH6*
MTA11*_7DAI	11	27,121,054	7.3	C/A	58.1	86.2	

*^a^* NM represent nymph mortality.

**Table 3.** QTL detected in F_2_ population derived from the cross between CC125 (resistance) and T65 (susceptible)

QTL	Marker	Chr.	Position (bp)	Peak LOD	Additive effect*^a^*	Dominance effect*^b^*	PVE*^c^* (%)	LOD threshold*^d^*
*qGRH11*	MTA11Id1	11	27,177,381	9.7	33.5	18.7	43.7	2.6

*^a^* Calculated as (X – Y)/2 where X and Y are trait values of CC125 homozygous and T65 homozygous plants, respectively. *^b^* Calculated as Z – (X + Y)/2, where Z is the trait value of heterozygous plants. *^c^* Percentage of explained phenotypic variation. *^d^* A LOD threshold of at 1% significant level.

## References

[B1] Bharathi, Y., V.D. Reddy, K.V. Rao and I.C. Pasalu (2018) Effect of mannose specific lectins *ASAL* and *GNA* on the feeding behavior of BPH (*Nilaparvata lugens* Stal.). Int J Curr Microbiol App Sci 7: 1426–1436.

[B2] Bhat, J.A., S. Ali, R.K. Salgotra, Z.A. Mir, S. Dutta, V. Jadon, A. Tyagi, M. Mushtaq, N. Jain, P.K. Singh et al. (2016) Genomic selection in the *era* of next generation sequencing for complex traits in plant breeding. Front Genet 7: 221. 28083016 10.3389/fgene.2016.00221 PMC5186759

[B3] Bolger, A.M., M. Lohse and B. Usadel (2014) Trimmomatic: A flexible trimmer for Illumina sequence data. Bioinformatics 30: 2114–2120. 24695404 10.1093/bioinformatics/btu170 PMC4103590

[B4] Brar, D.S., P.S. Virk, K.K. Jena and G.S. Khush (2009) Breeding for resistance to planthoppers in rice. *In*: Henong, K.L. and B. Hardy (eds.) Planthoppers: new threats to the sustainability of intensive rice production systems in Asia, International Rice Research Institute, Los Banos, Philippines, pp. 401–409.

[B5] Broman, K.W. and S. Sen (2009) A guide to QTL mapping with R/qtl. Springer, New York.

[B6] Calus, M.P., T.H. Meuwissen, J.J. Windig, E.F. Knol, C. Schrooten, A.L. Vereijken and R.F. Veerkamp (2009) Effects of the number of markers per haplotype and clustering of haplotypes on the accuracy of QTL mapping and prediction of genomic breeding values. Genet Sel Evol 41: 11. 19284677 10.1186/1297-9686-41-11 PMC3225874

[B7] Chang, X., M. Yang, H. Li, J. Wu, J. Zhang, C. Yin, W. Ma, H. Chen, F. Zhou and Y. Lin (2023) Cloning of the promoter of rice brown planthopper feeding-inducible gene *OsTPS31* and identification of related *cis*-regulatory elements. Pest Manag Sci 79: 1809–1819. 36637212 10.1002/ps.7356

[B8] Danecek, P., A. Auton, G. Abecasis, C.A. Albers, E. Banks, M.A. DePristo, R.E. Handsaker, G. Lunter, G.T. Marth, S.T. Sherry et al. (2011) The variant call format and VCFtools. Bioinformatics 27: 2156–2158. 21653522 10.1093/bioinformatics/btr330 PMC3137218

[B9] Dellaporta, S.L., J. Wood and J.B. Hicks (1983) A plant DNA minipreparation: Version II. Plant Mol Biol Report 1: 19–21.

[B10] De Vleesschauwer, D., G. Gheysen and M. Höfte (2013) Hormone defense networking in rice: tales from a different world. Trends Plant Sci 18: 555–565. 23910453 10.1016/j.tplants.2013.07.002

[B11] Dhatt, B.K., P. Paul, J. Sandhu, W. Hussain, L. Irvin, F. Zhu, M.A. Adviento-Borbe, A. Lorence, P. Staswick, H. Yu et al. (2021) Allelic variation in rice fertilization independent endosperm 1 contributes to grain width under high night temperature stress. New Phytol 229: 335–350. 32858766 10.1111/nph.16897 PMC7756756

[B12] Dimkpa, S.O.N., Z. Lahari, R. Shrestha, A. Douglas, G. Gheysen and A.H. Price (2016) A genome-wide association study of a global rice panel reveals resistance in *Oryza sativa* to root-knot nematodes. J Exp Bot 67: 1191–1200. 26552884 10.1093/jxb/erv470 PMC4753847

[B13] Du, B., W. Zhang, B. Liu, J. Hu, Z. Wei, Z. Shi, R. He, L. Zhu, R. Chen, B. Han et al. (2009) Identification and characterization of *Bph14*, a gene conferring resistance to brown planthopper in rice. Proc Natl Acad Sci USA 106: 22163–22168. 20018701 10.1073/pnas.0912139106 PMC2793316

[B14] Endelman, J.B. (2011) Ridge regression and other kernels for genomic selection with R package rrBLUP. Plant Genome 4: 250–255.

[B15] Eulgem, T., P.J. Rushton, S. Robatzek and I.E. Somssich (2000) The WRKY superfamily of plant transcription factors. Trends Plant Sci 5: 199–206. 10785665 10.1016/s1360-1385(00)01600-9

[B16] Fujino, K., M. Obara, T. Shimizu, K.O. Koyanagi and T. Ikegaya (2015) Genome-wide association mapping focusing on a rice population derived from rice breeding programs in a region. Breed Sci 65: 403–410. 26719743 10.1270/jsbbs.65.403 PMC4671701

[B17] Fujita, D., K. Doi, A. Yoshimura and H. Yasui (2004) Introgression of a resistance gene for green rice leafhopper from *Oryza nivara* into cultivated rice, *Oryza sativa* L. Rice Genet Newsl 21: 64–66.

[B18] Fujita, D., K. Doi, A. Yoshimura and H. Yasui (2006) Molecular mapping of a novel gene, *Grh5*, conferring resistance to green rice leafhopper (*Nephotettix cincticeps* Uhler) in rice, *Oryza sativa* L. Theor Appl Genet 113: 567–573. 16835766 10.1007/s00122-006-0270-x

[B19] Fujita, D., K. Doi, A. Yoshimura and H. Yasui (2010) A major QTL for resistance to green rice leafhopper (*Nephotettix cincticeps* Uhler) derived from African rice (*Oryza glaberrima* Steud.). Breed Sci 60: 336–341.

[B20] Fukuta, Y., K. Tamura, M. Hirae and S. Oya (1998) Genetic analysis of resistance to green rice leafhopper (*Nephotettix cincticeps* Uhler) in rice parental line, Norin-PL6, using RFLP markers. Japan J Breed 48: 243–249.

[B21] Furuta, T., O.M. Saw, S. Moe, K.T. Win, M.M. Hlaing, A.L.L. Hlaing, M.S. Thein, H. Yasui, M. Ashikari, A. Yoshimura et al. (2024) Development of genomic and genetic resources facilitating molecular genetic studies on untapped Myanmar rice germplasms Breed Sci 74: 124–137. 10.1270/jsbbs.23077 PMC11442107 39355624

[B22] Fuse, T., T. Sasaki and M. Yano (2001) Ti-plasmid vectors useful for functional analysis of rice genes. Plant Biotechnol 18: 219–222.

[B23] Ghauri, M.S.K. (1971) Revision of the genus *Nephotettix* Matsumura (Homoptera: Cicadelloidea: Euscelidae) based on the type material. Bull Entomol Res 60: 481–512.

[B24] Hamazaki, K. and H. Iwata (2020) RAINBOW: haplotype-based genome-wide association study using a novel SNP-set method. PLoS Comput Biol 16: e1007663. 32059004 10.1371/journal.pcbi.1007663 PMC7046296

[B25] Hayes, B.J., P.J. Bowman, A.C. Chamberlain, K. Verbyla and M.E. Goddard (2009) Accuracy of genomic breeding values in multi-breed dairy cattle populations. Genet Sel Evol 41: 51. 19930712 10.1186/1297-9686-41-51 PMC2791750

[B26] Henderson, C.R. (1975) Best linear unbiased estimation and prediction under a selection model. Biometrics 31: 423–447. 1174616

[B27] Hirae, M., Y. Fukuta, K. Tamura and S. Oya (2007) Artificial selection of biotypes of green rice leafhopper, *Nephotettix cincticeps* Uhler (Homoptera: Cicadellidae), and virulence to resistant rice varieties. Appl Entomol Zool 42: 97–107.

[B28] Hur, Y., J.H. Cho, J.Y. Lee, Y. Sohn, S. Park, B. Lee, J. Cho, S.Y. Kim, Y.C. Song, D. Park et al. (2015) Fine mapping of *GRH3* conferring resistance to green rice leafhopper in rice (*Oryza sativa* L.). Mol Breed 35: 89.

[B29] Jadamba, C., R.L. Vea, J.H. Ryu, N.C. Paek, S. Jang, J.H. Chin and S.C. Yoo (2022) GWAS analysis to elucidate genetic composition underlying a photoperiod-insensitive rice population, North Korea. Front Genet 13: 1036747. 36568369 10.3389/fgene.2022.1036747 PMC9768348

[B30] Ji, H., S.R. Kim, Y.H. Kim, J.P. Suh, H.M. Park, N. Sreenivasulu, G. Misra, S.M. Kim, S.L. Hechanova, H. Kim et al. (2016) Map-based cloning and characterization of the *BPH18* gene from wild rice conferring resistance to brown planthopper (BPH) insect pest. Sci Rep 6: 34376. 27682162 10.1038/srep34376 PMC5041133

[B31] Kadowaki, M., A. Yoshimura and H. Yasui (2003) RFLP mapping of antibiosis to rice green leafhopper. *In*: Khush, G.S., D.S. Brar and B. Hardy (eds.) Advances in rice genetics, International Rice Research Institute, Los Banos, Philippines, pp. 270–272.

[B32] Kaneda, C., M. Yokoo, H. Ikehashi, A. Kobayashi, R. Ikeda and H. Nemoto (1985) Breeding of rice Norin PL2, a new germplasm with resistance to green rice leafhopper and dwarf virus disease. Bull Natl Agric Res Cent 5: 81–91.

[B33] Kang, H., Y. Wang, S. Peng, Y. Zhang, Y. Xiao, D. Wang, S. Qu, Z. Li, S. Yan, Z. Wang et al. (2016) Dissection of the genetic architecture of rice resistance to the blast fungus *Magnaporthe oryzae*. Mol Plant Pathol 17: 959–972. 26574735 10.1111/mpp.12340 PMC6638458

[B34] Kang, H.M., N.A. Zaitlen, C.M. Wade, A. Kirby, D. Heckerman, M.J. Daly and E. Eskin (2008) Efficient control of population structure in model organism association mapping. Genetics 178: 1709–1723. 18385116 10.1534/genetics.107.080101 PMC2278096

[B35] Kawahara, Y., M. de la Bastide, J.P. Hamilton, H. Kanamori, W.R. McCombie, S. Ouyang, D.C. Schwartz, T. Tanaka, J. Wu, S. Zhou et al. (2013) Improvement of the *Oryza sativa* Nipponbare reference genome using next generation sequence and optical map data. Rice (N Y) 6: 4. 24280374 10.1186/1939-8433-6-4 PMC5395016

[B36] Ko, B.J., C. Lee, J. Kim, A. Rhie, D.A. Yoo, K. Howe, J. Wood, S. Cho, S. Brown, G. Formenti et al. (2022) Widespread false gene gains caused by duplication errors in genome assemblies. Genome Biol 23: 205. 36167596 10.1186/s13059-022-02764-1 PMC9516828

[B37] Kuang, H., S.S. Woo, B.C. Meyers, E. Nevo and R.W. Michelmore (2004) Multiple genetic processes result in heterogeneous rates of evolution within the major cluster disease resistance genes in lettuce. Plant Cell 16: 2870–2894. 15494555 10.1105/tpc.104.025502 PMC527186

[B38] Lee, H.A. and S.I. Yeom (2015) Plant NB-LRR proteins: tightly regulated sensors in a complex manner. Brief Funct Genomics 14: 233–242. 25825425 10.1093/bfgp/elv012

[B39] Mai, T.V., D. Fujita, M. Matsumura, A. Yoshimura and H. Yasui (2015) Genetic basis of multiple resistance to the brown planthopper (*Nilaparvata lugens* Stål) and the green rice leafhopper (*Nephotettix cincticeps* Uhler) in the rice cultivar ‘ASD7’ (*Oryza sativa* L. ssp. *indica*). Breed Sci 65: 420–429. 26719745 10.1270/jsbbs.65.420 PMC4671703

[B40] Maldonado, C., F. Mora, C.A. Scapim and M. Coan (2019) Genome-wide haplotype-based association analysis of key traits of plant lodging and architecture of maize identifies major determinants for leaf angle: *hap*LA4. PLoS One 14: e0212925. 30840677 10.1371/journal.pone.0212925 PMC6402688

[B41] McHale, L., X. Tan, P. Koehl and R.W. Michelmore (2006) Plant NBS-LRR proteins: adaptable guards. Genome Biol 7: 212. 16677430 10.1186/gb-2006-7-4-212 PMC1557992

[B42] Mizuno, H., S. Katagiri, H. Kanamori, Y. Mukai, T. Sasaki, T. Matsumoto and J. Wu (2020) Evolutionary dynamics and impacts of chromosome regions carrying *R*-gene clusters in rice. Sci Rep 10: 872. 31964985 10.1038/s41598-020-57729-w PMC6972905

[B43] Nakagome, M., M. Takimoto and Y. Uebayashi (1989) Insecticide susceptibility of the green rice leafhopper, *Nephotettix cincticeps* Uhler and resistance to insect of the rice breeding line Aichi 80. Res Bull Aichi Agric Res Cent 21: 78–84 (in Japanese with English summary).

[B44] Park, S.K., D.Y. Kwak, D.S. Park, D. Shin, W.H. Hwang, M.H. Kim, Y.J. Hur, J.H. Cho, Y.C. Song, J.Y. Lee et al. (2013) Fine mapping of *Grh1*, a major gene associated with antibiosis to green rice leafhopper in rice. Mol Breed 32: 729–733.

[B45] Pathak, M.D. and Z.R. Khan (1994) Insect pests of rice, International Rice Research Institute, Los Banos, Philippine, pp. 19–23.

[B46] Pathak, P.K. and E.A. Heinrichs (1982) Selection of biotype populations 2 and 3 of *Nilaparvata lugens* by exposure to resistant rice varieties. Environ Entomol 11: 85–90.

[B47] Phi, C.N., D. Fujita, Y. Yamagata, A. Yoshimura and H. Yasui (2019) High-resolution mapping of *GRH6*, a gene from *Oryza nivara* (Sharma et Shastry) conferring resistance to green rice leafhopper (*Nephotettix cincticeps* Uhler). Breed Sci 69: 439–446. 31598076 10.1270/jsbbs.19029 PMC6776147

[B48] Qiu, Y., J. Guo, S. Jing, L. Zhu and G. He (2012) Development and characterization of japonica rice lines carrying the brown planthopper-resistance gene *BPH12* and *BPH6*. Theor Appl Genet 124: 485–494. 22038433 10.1007/s00122-011-1722-5

[B49] Rahman, M.L., W. Jiang, S.H. Chu, Y. Qiao, T.H. Ham, M.O. Woo, J. Lee, M.S. Khanam, J.H. Chin, J.U. Jeung et al. (2009) High-resolution mapping of two rice brown planthopper resistance genes, *Bph20*(t) and *Bph21*(t), originating from *Oryza minuta*. Theor Appl Genet 119: 1237–1246. 19669727 10.1007/s00122-009-1125-z

[B50] Ronald, P.C. (1998) Resistance gene evolution. Curr Opin Plant Biol 1: 294–298. 10066604 10.1016/1369-5266(88)80049-9

[B51] Saka, N., T. Tsuji, T. Toyama, M. Yano, T. Izawa and T. Sasaki (2006) Development of cleaved amplified polymorphic sequence (CAPS) markers linked to a green rice leafhopper resistance gene, *Grh3(t)*. Plant Breed 125: 140–143.

[B52] Sebastian, L.S., R. Ikeda, N. Huang, T. Imbe, W.R. Coffman and S.R. McCouch (1996) Molecular mapping of resistance to rice tungro spherical virus and green leafhopper. J Phytopathol 86: 25–30.

[B53] Shi, S., H. Wang, L. Nie, D. Tan, C. Zhou, Q. Zhang, Y. Li, B. Du, J. Guo, J. Huang et al. (2021) *Bph30* confers resistance to brown planthopper by fortifying sclerenchyma in rice leaf sheaths. Mol Plant 14: 1714–1732. 34246801 10.1016/j.molp.2021.07.004

[B54] Shin, J., S. Blay, B. McNeney and J. Graham (2006) LDheatmap: an R function for graphical display of pairwise linkage disequilibria between single nucleotide polymorphisms. J Stat Softw 16: 1–9.

[B55] Stephens, M., N.J. Smith and P. Donnelly (2001) A new statistical method for haplotype reconstruction from population data. Am J Hum Genet 68: 978–989. 11254454 10.1086/319501 PMC1275651

[B56] Sun, L., C. Su, C. Wang, H. Zhai and J. Wan (2005) Mapping of a major resistance gene to the brown planthopper in the rice cultivar Rathu Heenati. Breed Sci 55: 391–396.

[B57] Takita, T. (1990) Gene analysis for resistance to green rice leafhoppers. Rice Genet Newsl 6: 117.

[B58] Tamura, K., Y. Fukuta, M. Hirae, S. Oya, I. Ashikawa and T. Yagi (1999) Mapping of the *Grh1* locus for green rice leafhopper resistance in rice using RFLP makers. Breed Sci 49: 11–14.

[B59] Tamura, K., Y. Fukuta, M. Hirae, S. Oya, I. Ashikawa and T. Yagi (2004) RFLP mapping of a new resistance gene for green rice leafhopper in Kanto PL10. Rice Genet Newsl 21: 62–64.

[B60] Tamura, Y., M. Hattori, H. Yoshioka, M. Yoshioka, A. Takahashi, J. Wu, N. Sentoku and H. Yasui (2014) Map-based cloning and characterization of a brown planthopper resistance gene *BPH26* from *Oryza sativa* L. ssp. indica cultivar ADR52. Sci Rep 4: 5872. 25076167 10.1038/srep05872 PMC5376202

[B61] Toki, S. (1997) Rapid and efficient *Agrobacterium*-mediated transformation in rice. Plant Mol Boil Report 15: 16–21.

[B62] Wang, X., Y. Xu, H. Fan, N. Cui, X. Meng, J. He, N. Ran and Y. Yu (2023) Research progress of plant nucleotide-binding leucine-rich repeat protein. Horticulture 9: 122.

[B63] Wilson, M.R. and M.F. Claridge (1985) The leafhopper and planthopper faunas of rice fields. *In*: Nault, L.R. and J.G. Rodriguez (eds.) The leafhoppers and planthoppers, John Wiley and Sons, New York, USA. pp. 381–404.

[B64] Würschum, T., H.P. Maurer, F. Dreyer and J.C. Reif (2013) Effect of inter- and intragenic epistasis on the heritability of oil content in rapeseed (*Brassica napus* L.). Theor Appl Genet 126: 435–441. 23052025 10.1007/s00122-012-1991-7

[B65] Yan, L., T. Luo, D. Huang, M. Wei, Z. Ma, C. Liu, Y. Qin, X. Zhou, Y. Lu, R. Li et al. (2023) Recent advances in molecular mechanism and breeding utilization of brown planthopper resistance genes in rice: an integrated review. Int J Mol Sci 24: 12061. 37569437 10.3390/ijms241512061 PMC10419156

[B66] Yang, H., A. You, Z. Yang, F. Zhang, R. He, L.L. Zhu and G.G. He (2004) High resolution genetic mapping at the *Bph15* locus for brown planthopper resistance in rice (*Oryza sativa* L.). Theor Appl Genet 110: 182–191. 15549231 10.1007/s00122-004-1844-0

[B67] Yasui, H. and A. Yoshimura (1999) QTL mapping of antibiosis to green leafhopper, *Nephotettix virescens* Distant and green rice leafhopper, *Nephotettix cincticeps* Uhler in rice, *Oryza sativa* L. Rice Genet Newsl 16: 96–97.

[B68] Yu, J., G. Pressoir, W.H. Briggs, I.V. Bi, M. Yamasaki, J.F. Doebley, M.D. McMullen, B.S. Gaut, D.M. Nielsen, J.B. Holland et al. (2006) A unified mixed-model method for association mapping that accounts for multiple levels of relatedness. Nat Genet 38: 203–208. 16380716 10.1038/ng1702

[B69] Zargar, S.M., B. Raatz, H. Sonah, MuslimaNazir, J.A. Bhat, Z.A. Dar, G.K. Agrawal and R. Rakwal (2015) Recent advances in molecular marker techniques: insight into QTL mapping, GWAS and genomic selection in plants. J Crop Sci Biotechnol 18: 293–308.

[B70] Zhang, P., K. Zhong, Z. Zhong and H. Tong (2019) Genome-wide association study of important agronomic traits within a core collection of rice (*Oryza sativa* L.). BMC Plant Biol 19: 259. 31208337 10.1186/s12870-019-1842-7 PMC6580581

[B71] Zhao, K., C.W. Tung, G.C. Eizenga, M.H. Wright, M.L. Ali, A.H. Price, G.J. Norton, M.R. Islam, A. Reynolds, J. Mezey et al. (2011) Genome-wide association mapping reveals a rich genetic architecture of complex traits in *Oryza sativa*. Nat Commun 2: 467. 21915109 10.1038/ncomms1467 PMC3195253

[B72] Zhao, Y., J. Huang, Z. Wang, S. Jing, Y. Wang, Y. Ouyang, B. Cai, X.F. Xin, X. Liu, C. Zhang et al. (2016) Allelic diversity in an NLR gene *BPH9* enables rice to combat planthopper variation. Proc Natl Acad Sci USA 113: 12850–12855. 27791169 10.1073/pnas.1614862113 PMC5111712

