# Supplementary material for: Resistance haplotypes to green rice leafhopper (Nephotettix cincticeps Uhler) estimated in genome-wide association study in Myanmar indica rice landraces
Source: Breed Sci. 2024 Aug 23;74(4):366–81. doi: 10.1270/jsbbs.23067 (PMC11769587; doi:10.1270/jsbbs.23067)
Supplement: Supplementary file 1 — Supplemental Figures [file 74_366-s1.pdf]

doi: 10.1270/jsbbs.23067

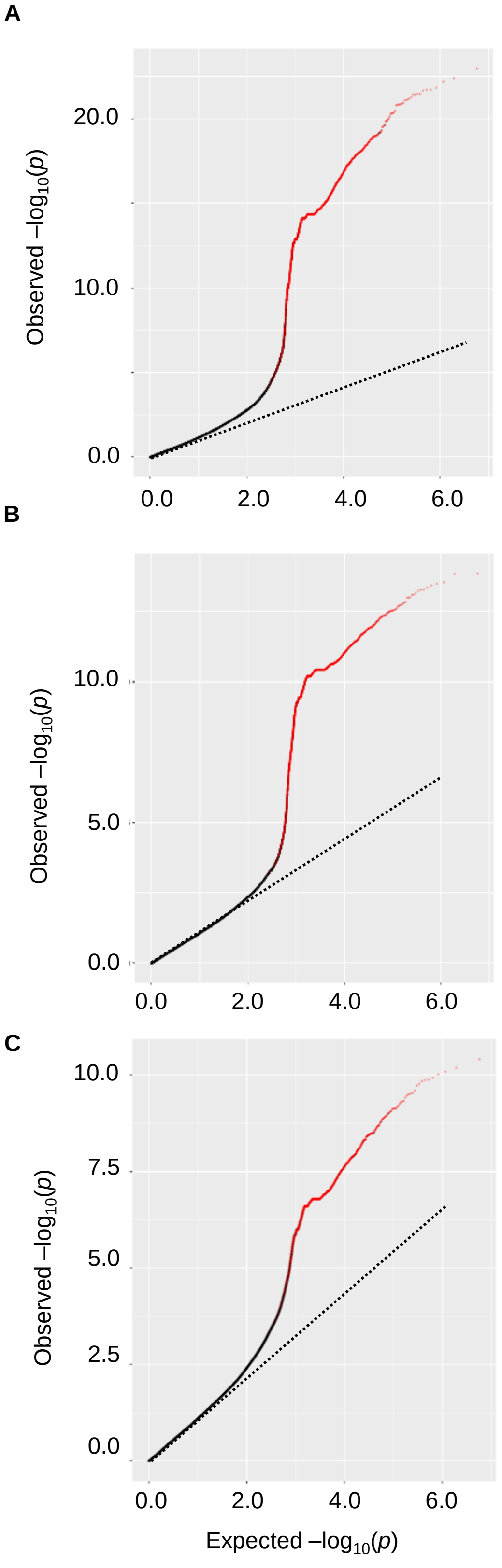

Supplemental Fig. 1. Quantile-quantile plots of the obtained *p* distribution in GWAS for nymph mortality with GRH resistance in Myanmar Indica diversity panel at (A) 3 days after infestation (DAI), (B) 5DAI, and (C) 7DAI. SNP markers in the MTA4, MTA5, and MTA11 regions are shown in red. The diagonal dotted lines represent the expected distribution under the null hypothesis, where there are no QTL.

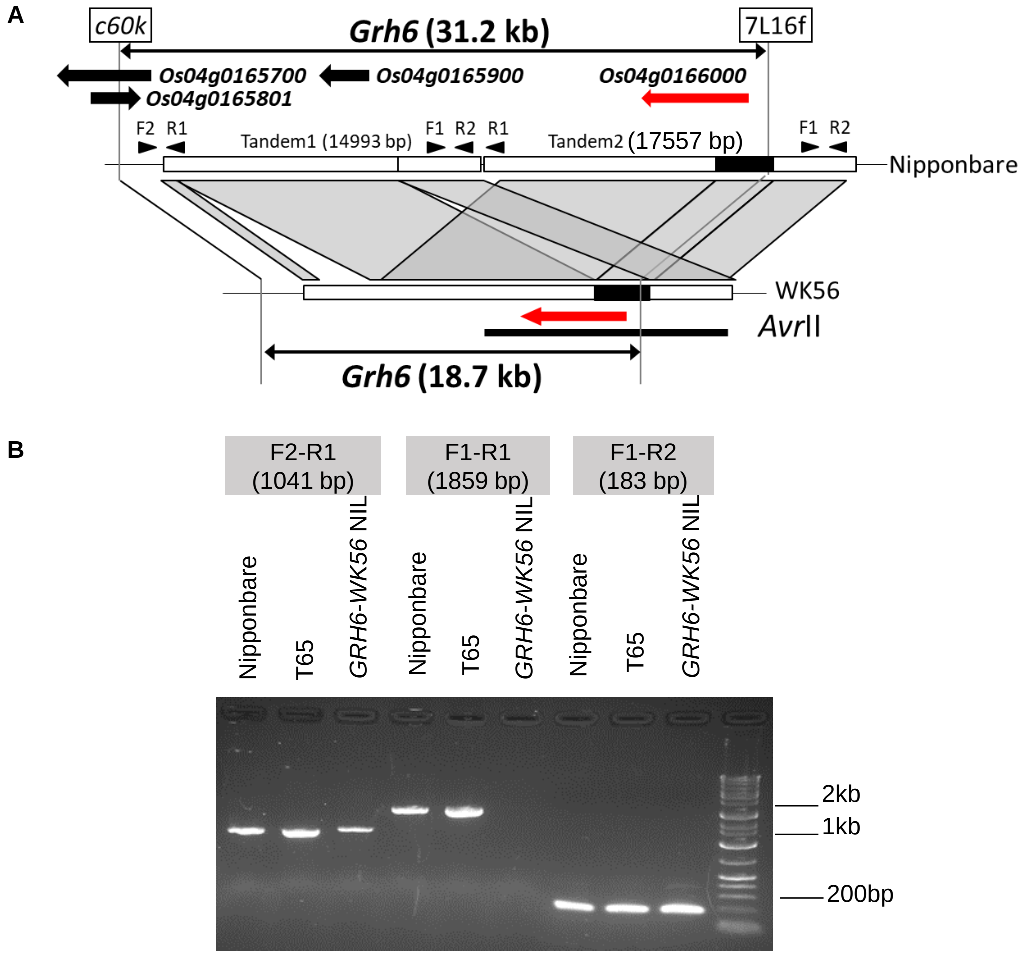

Supplemental Fig. 2. Confirmation of non-tandem duplication in WK56 at *GRH6* genomic region. (A) Genetic and physical position of *GRH6*. G6-c60k and 7L16f represents the flanking markers used in delimitation of *GRH6* region (Phi *et al*. 2019). The predicted genes annotated in the Rice Genome Annotation Project Database (RAP-DB) are shown as black and red arrows. The red arrow represents *Os04g0166000* encoding LRR protein and located on the tandemly duplicate region 2 (tandem2) in Nipponbare sequence. Primers for confirmation of the tandemly duplicated region 1 (tandem1) and tandem2 are shown in arrowheads. (B) PCR amplification from Nipponbare, T65, and *GRH6-WK56* NILs was successful using the primer pairs F2-R1 and F1-R2, suggesting that these primers were functional for the annealing and amplification of the PCR products. Nevertheless, no amplification was obtained from *GRH6-WK56* NIL, whereas PCR amplifications were obtained from Nipponbare and T65 using primers F1 and R1 owing to their tandem duplication. The results revealed that the tandem1 genomic region was deleted in WK56 and WK56 had only one copy of the gene, *Os04g0166000*, in the genomic region of *GRH6*.

```
GRH6-WK56     MRWKHINANSVKAVVKELIPYLEDTSSAARKSIYFEGWVGLGASAVLRAIAENPAPSLRK
GRH6-T65      MRRERIDATSVEAAVESLIPYLEDTSSAAHKSIYFDGWDGLGASAVLRAIAENPAPSLRK
              ** ::*:*.**:*.*:.************:*****:** *********************

GRH6-WK56     KFDRIIHVDCSRWKNPRQLQRAIADQLKLPQHVMDLFDRQDEEDDFSGVEESSRAGVTDI
GRH6-T65      KFDRIIHVDCSRWKKRRQLQRAIADQLKLPQHVMDLFDRQDEEDDFSGVEESSRAEVTDI
              **************: *************************************** ****

GRH6-WK56     GKEIYQTIKDLSCLLIFHNGSDDTVDTSKLGFPLYDWYNSNVNKVLWTFRGRLRLNPKIA
GRH6-T65      GKEIYQTIKDLSCLLIFHNGSDDTVDTSKLGFPLYDWSD---NKVLWTFRGRLRLNPKIA
              ************************************* :   ******************

GRH6-WK56     DNVDSSQLNIFSRHM-HF-SSYIWKDSLSREAEEITQYSGELHLDGTKAAECCLYLLSLN
GRH6-Nip      DNVDSSHLNIYSHYYYHFYSFESLNDLLPREAEEITQYSGELHLDGTKAAECCLYLLSLN
              ******:***:*::  ** *    :* * *******************************

GRH6-WK56     YWGGHIMDYNWATHACNYWVCDGIITVQEEKGGGSLQEDQAWELAAALHQEIRLEDYSSN
GRH6-Nip      YRGGHIMDYNWATHACNYWVCDGIITVQEEKGGGSLQEDQAWELAAALHQEIRLEDYSSN
              * **********************************************************

GRH6-WK56     RVPPFGYGLDTPPNRWVLVTQESDMKNKPTLDTTSLFIAFQSVVLLPDDMFHQANKVQVL
GRH6-Nip      RVPGFGHYLDTPPNRWVLVTQESDMKDKPTLETTSLFIAFQSVVLLPDDMFHQANKVQVL
              *** **: ******************:****:****************************

GRH6-WK56     RLCNCAFNFSLPPFHCCHNLRFLGLDKCQDHRTQEAGEDKSNNSSALEIFQRLWVLDICY
GRH6-Nip      RLCNCAFNFSSPPFHCCHNLRFLGLDKCQDHRTQEAGEDKSNTSSALEFFQRLWVLDICY
              ********** ********************************.*****:***********

GRH6-WK56     IDWELPFPTESTREQQMAMNIREVHINKGRIWRRNFAWRRLKNLRKLRVIEPTHPWGNKG
GRH6-Nip      IDWELPFPTESTREQQMAMNIREVHINKGRIWRRNFAWRRLKNLRKLRVIEPTHPWGNKG
              ************************************************************

GRH6-WK56     EIDEFADMLKLEILDLSKNTMIQVLPSLCGASSLKTLILDDCVVLEQVGPQGLPPSLESF
GRH6-Nip      EIDEFADMLKLEILDLSKNTMIQVLPSLCGASSLKTLILDDCVVLEQVGPQGLPPSLESF
              ************************************************************

GRH6-WK56     SFASREGNKAKISSISLAGCSSLVNFTLRGPLQNLRGLDLSGTMIKMLDLRDVQDSCIGQ
GRH6-Nip      SFASREGNKAKISSISLAGCSSLVNFTLRGPLQNLRGLDLSGTMIKMLDLRDVQDSCIGQ
              ************************************************************

GRH6-WK56     IILLRCEKLCTILWPGEGFPKLSMLHIDSLVCHVETEHQQAYATVMDLRFVQSLVLRSNY
GRH6-Nip      IILLRCEKLCTILWPEKGFPNLSMLHIDSLVCHVETEQQQAYATVMDLRFVQSLVLRSNN
              *************** :***:****************:********************* 

GRH6-WK56     NFCWNYNKTHINICISSTPKDATPKKKTMSYYSAQKVVGSPLHMPIVTTIQPVVCYKDVN
GRH6-Nip      KFCWNCNKTHINICISSTPKDATPKKKTMSYYSAQKVVGSPLHMPIVTTIQPVVCYKDVN
              :**** ******************************************************

GRH6-WK56     LAMISAIELEGSSAPRHEPLDIHVEIGEGISYANVVSEEAWSAVSFIMNEAESLHVHDNF
GRH6-Nip      LAMISAIELEGSSAPRHEPLDIHVEIGEGISYANVVSEQALSAVSFMMNKAKSLHVHDNF
              **************************************:* *****:**:*:********

GRH6-WK56     SITSVNPKHMMS----QVGWDCLKRCHIERCHRLNPVFFIDYIGTWFETLEAFSAAELMM
GRH6-Nip      SITSVNPKHVILTEDKEITWYCLKRCHIERCHKLNTVFSTDYIKYRFQTLEAFSAAELMM
              *********::     :: * ***********:** **  ***   *:************

GRH6-WK56     ANCIWSRGRTFPRSESNTFAKLRSIHLHYCPRLTFVLPLSWPTSDSHLPSLETLHIVYCS
GRH6-Nip      ANCIWSRGRTFPGWNSNMFAKLRSIHLHYCPRLTFVLPLSWPTPYSHLPSLETLHIVYCS
              ************  :** *************************  ***************

GRH6-WK56     ELRQIFPVEAVALREQPRGVLRFPKLKHIHLYDVPKLHQICEISRMVAPVLETIRVRGCW
GRH6-Nip      ELRQIFPVEAVALREQPRGVLRFPKLKHIHLHDVPKLHQICEISRMVAPVLETIRVRGCW
              *******************************:****************************

GRH6-WK56     ALKRIPAIDGSLRGQDSRPIVNCEKDWWEKLEWEGMNVGHDPSLFEPRHSMYYKKALPRC
GRH6-Nip      ALKRIPAING-------RPIVDCEKDWWEKLEWEGMNVGHDPSLFEPRHSMYYKKALPRC
              ********:*       ****:**************************************

GRH6-WK56     SLLR*
GRH6-Nip      SLLR*
              *****
```

Supplemental Fig. 3. Amino acid sequence comparison of *GRH6-WK56* between WK56 and Nipponbare. Blue letters represent the LRR domain predicted in Interpro database (IPR032675).

```
Tandem2T65      ACAACAATAGAAAATTAAGATAACACCACCAATACCTAAGTATTGAACTCTGATCGTGCG
WK56            ACAACAATAGAAAATTAAGATAACACCACCAATACCTAAGTATTGAACTCTGATCGTGCG
                ************************************************************

Tandem2T65      TGCAGGAATATAATAATCCTAATAATCCGGCACCGGTGCCTAATGATCTCTGTTACCTGG
WK56            TGCAGGAATATAATAATCCTAATAATCCGGCACCGGTGCCTAATGATCTCTGTTACCTGG
                ************************************************************

Tandem2T65      ATTGAACCGAGGAGAAAAAATGTTGTCTCAATTCCTTTTCTTCCTTGGCCGTCAAGGTAG
WK56            ATTGAACCGAGGAGAAAAAATGTTGTCTCAATTCCTTTTCTTCCTTGGCCGTCAAGGTAG
                ************************************************************

Tandem2T65      CATTATCATCTACTGATTTACAACCTCCTTAATTAACAAGCACGGGTTTCATGTCAACAG
WK56            CATTATCATCTACTGATTTACAACCTCCTTAATTAACAAGCACGGGTTTCATGTCAACAG
                ************************************************************

Tandem2T65      AAAATTGCCGTTTAGCCATGGACATAACTTGTGAAAAAGAAACTTGATAATAGGTGGTGT
WK56            AAAATTGCCGTTTAGCCATGGACATAATTTGTGAAAAAGAAACTTGATAATAGGTGGTGT
                *************************** ********************************

Tandem2T65      AAAATTGTGACAGTACATGGTATATAAATTGTTATGTTCCAAACATATAAATGACTTTTC
WK56            AAAATTGTGACAGTACATGGTATATAAATTGTTATGTTCCAAACATAGAAATGACTTTTC
                *********************************************** ************

Tandem2T65      CAATATTGAGATTTATAATAATAACTACATATATGGCTGTGTTCTTTTCCCCTCTTTC--
WK56            CAATATTGAGATTTATAATAATAACTACATATATGGCTGTGTTCTTTTCCCCTCTTTCCT
                **********************************************************

Tandem2T65      AACTCACTTTCATTGTTTCCGCGCACACGCTTTTCAAACTACTGAACGGTTTACTTTTTG
WK56            AACTCACTTTCATTGTTTCCGCGCACACGCTTTTCAAACTACTAAACGGTTTACTTTTTG
                ******************************************* ****************

Tandem2T65      CAAAAGTTTTCTATATGAAAGTTGCTTTAAAAAAAATCATATTAATCCATTTTTCAAGCT
WK56            CAAAAGTTTTCTATATGAAAGTTGCTTTAAAAAAA-TCATATTAATCCATTTTTCAAGCT
                *********************************** ************************

Tandem2T65      TTTTAGACTAATGCTTAATTAATCTCAATATAATCGCGTGCTTTGTTTTCCGTGTCAGGA
WK56            TTTTAGACTAATGCTTAATTAATCTCAATATAATCGCGTGCTTTGTTTTCCGTGTCAAGA
                ********************************************************* **
                                                   7L16F Reverse primer =>
Tandem2T65      GGCAAGGTGGGAAACTTGCCCTCCCGAACACAGCCCTGGGTTGCAAATGCACGACACATA
WK56            GGCAATGTTGGAAACTTGCCCTCCCGAACACAGCCCTGGGTTGCAAATGCACGACACATA
                ***** ** ***************************************************

Tandem2T65      AATGATTGAGATCAAAGCATTAAAAAGATCCAAAAACTAATATTTTCTTGATGATGTGGC
WK56            AATGATTGAGATCAAAGCATTAAAAAGATCCAAAAACTAATATTTTCTTGATGATGTGGC
                ************************************************************

Tandem2T65      TCTATGCATGTCAAGCTTTTGATTCTAGTAACTATACTAAGTAGGTTGTATGAATTCGGG
WK56            TCTATGCATGTCAAGCT---------AGTAACTATACTAAGTAGGTTGTATGAATTCGGG
                *****************         **********************************

Tandem2T65      TTCAGGCGGGTTAGGTGTGATACTGAGGAATAGTTCTGGCAAACCCATTTTTTCTGCTTG
WK56            TTCAGGCGGGTTAGGTGTGATACTGAGGAATAGTTCTGGCAAACCCATTTTTTCTGCTTG
                ************************************************************

Tandem2T65      CGGATTCATCGAGCGCTGCAGTAGCCCTTTGGAAGCTGAACTACTAGCCTGCAAAGAGGG
WK56            CGGATTCATCGAGCGCTGCAGTAGCCCTTTGGAAGCTGAACTACTAGCCTGCAAAGAGGG
                ************************************************************

Tandem2T65      CATTATTATGGCCCTT----GGACCTTGCTGCCCATCATTGTGGAGTTTGATTGCTCGGT
WK56            CATTATTATGGCCCTTCAGTGGACCTTGCTGCCCATCATTGTGGAGTTTGATTGCTCGGT
                ****************    ****************************************

Tandem2T65      CGCAGTGAATATGATCCAGGCTGCGATGGAGGAGAAGTCTCAGTTTGCCCATCTGACACG
WK56            AGCAGTGAATATGATCCAGGCTGCGATGGAGGAGAAGTCT--------------------
                ****************************************

Tandem2T65      GGACATAGGAAACCTGATCACAGGAAACAGGGAAGTTTTACAACGGAAATCAATCGTACT
WK56            ------------------------------------------------------------

                <= 7L16f forward primer
Tandem2T65      CAGAACCGCATTAGCCATTTCCTAGCCAATAAGGCCCGTGCTGAACTCTGTATAGAGTTT
WK56            CAGAACCGCATTAGCCATTTCCTAGCCAATAAGGCCCGTGCTGAACTCTGTATAGAGTTT
                ************************************************************
                                  (1)    (2)
Tandem2T65      TGGCCAGACGAAAATTGTGATATTAACTCACAATTTGTTTGTGAGGAGGCTGCTTCCGAG
WK56            TGGCCAGACGAAAATTGTAATATTATCTCACAATTTGTTTGTGAGGAGGCTGCTTCCGAG
                ****************** ****** **********************************
```

Supplemental Fig. 4. *Continued on next page.*

```
                          (3)    (4) (5)        (6)
Tandem2T65      TAATATATTCTCTTTTCCCCGAAAAAAAATACTAAGTAGGTTGTAAATGTACATGATATA
WK56            AAATATATTCCCTTTTTCCTGAAAAAAA--ACTAAGTAGGTTGTAAATGTACATGATATA
                 ********* ***** ** ********  ******************************

                                                           (7)
Tandem2T65      TAAATTGTTGAGATCAAAGCATGAGAAATATGCAAAATCTAAT-TTTTTTTTTATGATGT
WK56            TAAATTGTTGAGATCAAAGCATGAGAAATATGCAAAATCTAATATTTTTTTTTATGATGT
                ******************************************* ****************

                                      (8)                                (9)
Tandem2T65      GGCTCTTTGCATGCATGTCAACATTTTTCTCCAACTATTAAAATATATAGATTGGATACG
WK56            GGCTCTTTGCATGCATGTCAACCTTTTTCTCCAACTATTAAAATATATAGATTGGATAGG
                ********************** *********************************** *

Tandem2T65      ATACATGATAAGTGATGGCACTATCGATATCCATACCCGGCCCCAACTGAAGAACAATAC
WK56            ATACATGATAAGTGATGGCACTATCGATATCCATACCCGGCCCCAACTGAAGAACAATAC
                ************************************************************

Tandem2T65      GATATACTGTGATTTGAACCTGTTCCAGCTCTGAGGAAAAATATGAAATAGGATACAAGA
WK56            GATATACTGTGATTTGAACCTGTTCCAGCTCTGAGGAAAAATATGAAATAGGATACAAGA
                ************************************************************

                          (10)                       (11)
Tandem2T65      ACGTGATATGACCGGACATGACCTGATTTCATCTCTATATAGAGATAAAGAAGTTGTGTA
WK56            ACGTGATATGCCCGGACATGACCTGATTTCATCTCTATCTAGAGATAAAGAAGTTGTGTA
                ********** *************************** *********************

Tandem2T65      AAATTACCGCGTCTAGTGATGCGAAGAAGTTACTCCCCTGAAATAACTGAGTAGATTATC
WK56            AAATTACCGCGTCTAGTGATGCGAAGAAGTTACTCCCCTGAAATAACTGAGTAGATTATC
                ************************************************************

Tandem2T65      GGCTTTGGATGTAATGAATTCACGTTGGAGCCATATGTATATATCGATGAGTCATCGATC
WK56            GGCTTTGGATGTAATGAATTCACGTTGGAGCCATATGTATATATCGATGAGTCATCGATC
                ************************************************************

                                                   (12)
Tandem2T65      GAGCTCTTGCAAAACGGATATGTAGACTAATGGTTGTAGTGACCTAAGTAGCACTCTAAG
WK56            GAGCTCTTGCAAAACGGATATGTAGACTAATGGTTGCAGTGACCTAAGTAGCACTCTAAG
                ************************************ ***********************

Tandem2T65      GTTCTGAGTTTAAATCTTTATATGAGTGAATTTCACATTAGGTTGTTTGATAGGCTAAGT
WK56            GTTCTGAGTTTAAATCTTTATATGAGTGAATTTCACATTAGGTTGTTTGATAGGCTAAGT
                ************************************************************

                  (13)                                      (14)
Tandem2T65      TCCTAATTTAAAAAGGCTGCATATATCCGGTTGGATGTAGAGACCGGGCAAAATATACCC
WK56            TCTTAATTTAAAAAGGCTGCATATATCCGGTTGGATGTAGAGGCCGGGCAAAATATACCC
                ** *************************************** *****************

                            (15)
Tandem2T65      TTCTCTAAAAAAATAAAAAATCGATTGAGCTCTTTTTTTTTTCTTTTCTTCTACCTTTCA
WK56            TTCTCTAAAAAA-TAAAAAATCGATTGAGCTCTTTTTTTTTTCTTTTCTTCTACCTTTCA
                ************ ***********************************************

Tandem2T65      TCTTTATTCGTTAGGTGTGTCCCAAGCAAAGAGCAAAGCATTGGATATATTCCTTTATGA
WK56            TCTTTATTCGTTAGGTGTGTCCCAAGCAAAGAGCAAAGCATTGGATATATTCCTTTATGA
                ************************************************************

Tandem2T65      AAGGCAATGCCAAAACGAAACATGAAGATAGCACAATTGAGTAGCTACACTACGCATCAA
WK56            AAGGCAATGCCAAAACGAAACATGAAGATAGCACAATTGAGTAGCTACACTACGCATCAA
                ************************************************************

                                 (16)                                  →Transcription
Tandem2T65      AGTGCTTATTTAATTC---------GCTAGGTAGTTCCCTGGCCTTGACGATCA    start (+1)
WK56            AGTGCTTATTTAATTCAGATGTGCAGCTAGGTAGTTCCCTGGCCTTGACGATCA
                ****************         *****************************
```

Supplemental Fig. 4. The promoter sequences of *GRH6-WK56* were derived from WK56 and a tandem 2 copy of T65. The location of the insertion/deletion marker, 7L16f, used for the fine mapping of *GRH6* (Phi *et al.* 2019) are shown in blue. Sixteen possible causative polymorphisms that may be involved in the low or non-expression of T65 allele, are marked in parentheses.
